# Collective effects in an incompressible electronic liquid

**DOI:** 10.1093/nsr/nwac251

**Published:** 2022-10-23

**Authors:** Jian-Jian Miao, Hui-Ke Jin, Yi Zhou

**Affiliations:** Department of Physics, The Chinese University of Hong Kong, Hong Kong, China; Department of Physics TQM, Technische Universität München, Garching D-85748, Germany; Institute of Physics, Chinese Academy of Sciences, Beijing 100190, China; Songshan Lake Materials Laboratory, Dongguan 523808, China; Kavli Institute for Theoretical Sciences & CAS Center for Excellence in Topological Quantum Computation, University of Chinese Academy of Sciences, Beijing 100190, China

**Keywords:** quantum spin liquid, Landau-type effective theory, collective modes, zero sound, first sound

## Abstract

Starting from Landau’s kinetic equation, we show that an electronic liquid in *d* = 2, 3 spatial dimensions depicted by a Landau-type effective theory will become incompressible on condition that the Landau parameters satisfy either (i) $1+F_{1}^{s}/d=0$ or (ii) $F_{0}^{s}\rightarrow +\infty$. Condition (i) is the Pomeranchuk instability in the current channel and suggests a quantum spin liquid (QSL) state with a spinon Fermi surface; while condition (ii) means that the strong repulsion in the charge channel leads to a conventional charge and thermal insulator. In the collisionless regime (ωτ ≫ 1) and the hydrodynamic regime (ωτ ≪ 1), the zero and first sound modes have been studied and classified by symmetries, including the longitudinal and transverse modes in *d* = 2, 3 and the higher angular momentum modes in *d* = 3. The sufficient (and/or necessary) conditions of these collective modes have been revealed. It has been demonstrated that some of these collective modes will behave in quite different manners under incompressibility condition (i) or (ii). Possible nematic QSL states and a hierarchy structure for gapless QSL states have been proposed in *d* = 3.

## Teaser

Zero and first sound modes in a gapless quantum spin liquid have been studied by using a Landau type effective theory, which is beyond the usual U(1) gauge theory.

## INTRODUCTION

The past few decades have witnessed a proliferation of research interest in quantum spin liquids (QSLs). As a novel phase of matter, QSL was proposed as a pristine Mott insulator that carries an odd number of electrons per unit cell, preserves the lattice translational symmetry and hosts a paramagnetic ground state [1–8]. Such a ‘featureless’ Mott insulator is characterized by fractional spin excitations and long-range quantum entanglement, which are beyond Landau’s symmetry breaking paradigm and naturally associated with the celebrated idea of the resonating valence bond (RVB) [1]. Moreover, high-temperature superconductivity has been considered to arise from doping such an RVB state [2,9].

While a decisive experiment for identifying a QSL state is still missing, this exotic phase of matter has been well established theoretically via exactly solvable models together with other analytical and numerical analyses [5–8]. Mathematically, the Lieb-Schultz-Mattis-Oshikawa-Hastings theorem [10–13] states that, when the lattice translational symmetry and the *O*(2) spin rotation conservation are preserved, the ground state of a quantum spin system is either gapless or gapped with topological degeneracy, if the *O*(2) charge (i.e., the spin quantum) per unit cell is a half-odd integer. This imposes a strong constraint on QSL ground states and low-energy excitations on top of them: a QSL state is either gapped with topological order, or gapless. In contrast to gapped QSLs that have been extensively studied by exploiting topological orders and various numerical tools, systematic studies on gapless QSLs are relatively rare.

On the experiment side, a variety of QSL candidates has been found in realistic materials, and many, if not most of them, exhibit gapless features [5,8]. Indeed, gapless QSLs are expected to display many-body electronic excitations inside the Mott charge gap and manifest themselves in various experiments that include but are not limited to thermodynamic and magnetic measurements, thermal transports, neutron scattering, nuclear magnetic resonance, muon spin resonance (μSR), optical conductivity, Raman scattering, the thermal Hall effect, sound attenuation and angle-resolved photo-emission spectroscopy [5].

Most theoretical studies on QSLs and/or relevant metal-to-Mott-insulator transitions start with a Mott insulator, while the dual approaches from the metallic side are relatively less known [14,15]. On the other hand, metallic states have been well understood by using the classic Landau’s Fermi liquid theory [16–18]. Collective modes in a Fermi liquid represent a class of elementary excitations, which involves a coherent motion of the system as an entirety. In this work, we study electronic collective modes in a gapless QSL state with a spinon Fermi surface that are naturally conveyed from a quasiparticle Fermi surface in an incompressible (i.e., insulating) limit. In particular, we focus on density fluctuations of chargeful quasiparticles on the Fermi surface, and study these collective modes in the gapless QSL state in three dimensions and two dimensions.

### An effective theory for QSL: revisit

Landau’s Fermi liquid theory describes a system of interacting fermions, which evolves from a non-interacting Fermi gas in a continuous way, namely, there is no phase transition as long as fermion-fermion interactions are turned on adiabatically. Thus, the low-energy excited states of interacting fermions in the Fock space remain one-to-one correspondence to those of a non-interacting Fermi gas, which are called ‘quasiparticles’ and labeled by fermion occupation numbers. Landau’s Fermi liquid theory is built with these quasiparticles and interactions between them [16–18].

An effective theory was proposed for gapless QSLs with a spinon Fermi surface in [15], which takes almost the same form as Landau’s Fermi liquid theory. The building blocks in effective QSL theory are assumed to be ‘chargeful’ quasiparticles, even though these quasiparticles are *not* protected by adiabaticity. The quasiparticles in these QSLs are labeled by the same occupation numbers as free fermions. Consequently, by defining $\delta n_{\mathrm{p\sigma }}=n_{\mathrm{p\sigma }}-n_{\mathrm{p\sigma }}^{0}$, the departure of the fermion distribution function from the ground-state distribution $n_{\mathrm{p}}^{0}=\theta (-\xi _{\mathrm{p}})$, the excitation energy Δ*E* = *E* − *E_G_* for these states are also given by a Landau-type expression:


(1) \begin{eqnarray*} \Delta E &=& \sum _{\mathrm{p\sigma }}\xi _{\mathrm{p}}\delta n_{\mathrm{p\sigma }}+\frac{1}{2}\sum _{\mathrm{pp}^{\prime }\sigma \sigma ^{\prime }}f_{\mathrm{pp}^{\prime }}^{\sigma \sigma ^{\prime }}\delta n_{\mathrm{p}\sigma }\delta {}n_{\mathrm{p}^{\prime }\sigma ^{\prime }} \\ && +\, O(\delta n^{3}). \end{eqnarray*}

Here ξ_p_ = p^2^/2m* − μ is the single-particle energy measured from the chemical potential μ, m* is the effective mass, σ (σ′) is the spin index and $f_{\mathrm{pp}^{\prime }}^{\sigma \sigma ^{\prime }}$ is the interaction energy between excited quasiparticles. Then the single quasiparticle excitation energy reads


(2) \begin{eqnarray*} \varepsilon _{\mathrm{p\sigma }}\equiv \frac{\delta {}\Delta {}E}{\delta (\delta {}n_{\mathrm{p}\sigma })}=\frac{\mathrm{p}^{2}}{2m^{\ast }} -\mu + \sum _{\mathrm{p}^{\prime } \sigma ^{\prime }}f_{\mathrm{pp}^{\prime }}^{\sigma \sigma ^{\prime }}\delta n_{\mathrm{p}^{\prime }\sigma ^{\prime }}.\\ \end{eqnarray*}

Here a rotational symmetry is assumed for the Fermi surface, and $f_{\mathrm{pp}^{\prime }}^{\sigma \sigma ^{\prime }}$ can be written in terms of spin symmetric and anti-symmetric components $f_{\mathrm{pp}^{\prime }}^{\sigma \sigma ^{\prime }}=f_{\mathrm{pp}^{\prime }}^{s}\delta _{\sigma \sigma ^{\prime }}+f_{\mathrm{pp}^{\prime }}^{a}\sigma \sigma ^{\prime }$, where $f_{\mathrm{pp}^{\prime }}^{s(a)}$ depends only on the angle θ between p and p′. It can be expanded as


(3a) \begin{eqnarray*} f_{\mathrm{pp}^{\prime }}^{s(a)}=\sum _{l=0}^{\infty }f_{l}^{s(a)}P_{l}(\cos \theta ) \end{eqnarray*}

with the *P_l_* the Legendre polynomials in three dimensions, or


(3b) \begin{eqnarray*} f_{\mathrm{pp}^{\prime }}^{s(a)}=\sum _{l=0}^{\infty }f_{l}^{s(a)}\cos (l\theta ), \end{eqnarray*}

in two dimensions. The dimensionless Landau parameters, defined by $F_{l}^{s(a)}=N(0)f_{l}^{s(a)}$, provide measures of the strengths of the interactions between quasiparticles, where *N*(0) is the Fermi surface density of states. The low-temperature properties of the QSLs are completely determined by the effective mass m* and the Landau parameters $F_{l}^{s(a)}$ as m* and $F_{l}^{s(a)}$ do in the Landau's Fermi liquid theory.

Landau’s Fermi liquid theory for interacting electrons describes a metallic state naturally. Thus, extra constraints have to be imposed to ensure that elementary excitations in the effective QSL theory carry zero charge and finite entropy, whereas quasiparticles are chargeful. It turns out that an electrical insulating but simultaneously thermal conducting state, namely, a QSL with a spinon Fermi surface, can be achieved by putting a constraint on the Landau parameter $F_1^s$. This can be seen as follows. First, the charge current $\mathbf {J}$ carried by quasiparticles is given by


(4a) \begin{eqnarray*} \mathbf {J}=\frac{m}{m^{\ast }}\bigg (1+\frac{F_{1}^{s}}{d}\bigg )\mathbf {J}^{(0)}, \end{eqnarray*}

where $\mathbf {J}^{(0)}$ is the charge current carried by the corresponding non-interacting fermions and *d* is the dimensionality. Meanwhile, the thermal current $\mathbf {J}_{Q}$ is only renormalized by the effective mass and reads


(4b) \begin{eqnarray*} \mathbf {J}_{Q}=\frac{m}{m^{\ast }}\mathbf {J}_{Q}^{(0)}, \end{eqnarray*}

where $\mathbf {J}_{Q}^{(0)}$ is the corresponding thermal current carried by non-interacting electrons. Therefore, when


(4c) \begin{eqnarray*} 1+\frac{F_{1}^{s}}{d}\rightarrow 0\quad \mbox{and}\quad \frac{m^{\ast }}{m}\ne {}0, \end{eqnarray*}

we have $\mathbf {J}\rightarrow 0$ and $\mathbf {J}_{Q}\ne 0$, suggesting that the electronic system is in a special state where spin-1/2 quasiparticles do not carry charge due to interaction but they still carry entropy.

Two issues have to be clarified. First, it is crucial that $F_{1}^{s}$ is independent of m*/*m* for such a mechanism to work. Indeed, it cannot happen in a Galilean invariant system, where ${m^{\ast }}/{m}=1+{F_{1}^{s}}/{d}$; therefore, the charge carried by quasiparticles does not vary with $F_{1}^{s}$. Fortunately, there does exist a way out for electrons in crystals, where the Galilean invariance is lost, so that ${m^{\ast }}/{m}\ne 1+{F_{1}^{s}}/{d}$ and $\mathbf {J}$ is renormalized by interactions. Second, the quasiparticles should be distinguished from elementary excitations in the effective theory. The quasiparticles are chargeful and described by δ*n*_*p*σ_, whereas elementary excitations are eigenstates of Landau’s kinetic equation with charges renormalized by $1+F_{1}^{s}/d$ and become zero in the limit of q = 0 and ω = 0, where q and ω are the wave vector and frequency, respectively.

### Landau’s kinetic equation and collective modes

Landau’s kinetic equation, which is more than a Boltzmann equation, can be utilized to study both equilibrium and non-equilibrium properties for a quantum liquid of fermions. It describes the change of the quasiparticle distribution function *n*_pσ_(r, *t*) in the phase space,


(5) \begin{eqnarray*} &&\frac{\partial n_{\mathrm{p}}(\mathrm{r},t)}{\partial {}t}+\nabla _{\mathrm{p}}\varepsilon _{\mathrm{p}}(\mathrm{r},t)\cdot \nabla _{\mathrm{r}}n_{\mathrm{p}}(\mathrm{r},t)-\nabla _{\mathrm{r}}\varepsilon _{\mathrm{p}}(\mathrm{r},t) \\ &&\quad\quad \cdot \nabla _{\mathrm{p}}n_{\mathrm{p}}(\mathrm{r},t)=I[n_{\mathrm{p}^{\prime }}], \end{eqnarray*}

where $I[n_{\mathrm{p}^{\prime }}]$ is the collision integral. This equation is considerably richer than the usual Boltzmann equation used to study weakly interacting gas, because ‘quasiparticles’ are not bare particles. Instead, they are combined with characteristics from the background, say, other particles in the quantum fluid. Thus, Landau’s kinetic equation allows us to study collective effects in such a quantum fluid.

Collective modes are the coherent motions of the quasiparticles on the Fermi surface. For the charge sector, the collective modes are characterized by the density fluctuation δ*n*_p_, which can be written as


(6) \begin{eqnarray*} \delta n_{\mathrm{p}}=-\frac{\partial n_{\mathrm{p}}^{0}}{\partial \varepsilon _{\mathrm{p}}}\nu _{\mathrm{p}}. \end{eqnarray*}

Here ν_p_ is the energy by which the density wave shifts the quasiparticle distribution in the direction of p, and can be expanded in terms of spherical harmonics, i.e.,


(7) \begin{eqnarray*} \nu _{\mathrm{p}}=\sum _{l}\sum _{m=-l}^{l}Y_{l}^{m}(\theta _{\mathrm{p}},\phi _{\mathrm{p}})\nu _{l}^{m}, \end{eqnarray*}

in three dimensions, or in terms of a Fourier series, i.e.,


(8) \begin{eqnarray*} \nu _{\mathrm{p}}=\sum _{l=-\infty }^{\infty }\nu _{l}e^{i{}l\theta _{\mathrm{p}}}, \end{eqnarray*}

in two dimensions. For later use, we also define $\nu _{l}=\nu _{l}^{0}$ in three dimensions. As long as the propagation wave vector q is specified, the azimuthal angle θ_p_ and polar angle φ_p_ can be defined with respect to the direction of $\mathrm{\hat{q}}$.

In this work, we are particular interested in two types of collective mode: zero sound in the collisionless regime, ωτ ≫ 1, and first sound in the hydrodynamic regime, ωτ ≪ 1.

## RESULTS

### Symmetry classification of collective modes

The collective modes can be classified in accordance with symmetries. There is a significant difference between two and three dimensions: a spherical Fermi surface has *O*(3) rotational symmetry, while a circular Fermi surface is of *O*(2) symmetry only. In the presence of a collective mode with a specified propagation wave vector q that breaks *O*(3) or *O*(2) symmetry, the symmetry of Landau’s kinetic equation will be reduced to the *O*(2) rotation along q in three dimensions or the *Z*_2_ reflection about q, θ_p_ → − θ_p_, in two dimensions.

**Figure 1.**
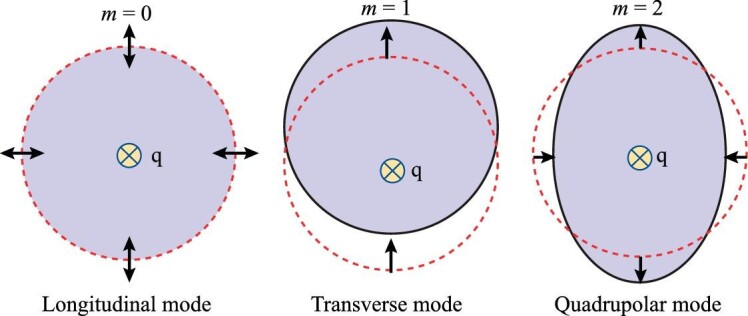
Collective modes in three dimensions. The propagation wave vector q is normal to the plane.

For three dimensions, the *O*(2) rotational symmetry gives rise to the conservation of the magnetic quantum number *m* defined in (7). The collective modes in three dimensions can then be classified in accordance with *m* as follows: ‘longitudinal’ (*m* = 0), ‘transverse’ (*m* = 1), ‘quadrupolar’ (*m* = 2) and other higher angular momentum (*m* ≥ 2) modes. These collective modes are illustrated in Fig. 1.

For two dimensions, the *Z*_2_ reflection symmetry can be manifested by rewriting (8) in terms of real components,


(9) \begin{eqnarray*} \nu _{\mathrm{p}}=\nu _{0}+\sum _{l=1}^{\infty }u_{l}\cos {}(l\theta _{\mathrm{p}})+\sum _{l=1}^{\infty }v_{l}\sin {}(l\theta _{\mathrm{p}}), \\ \end{eqnarray*}

where *u*_0_ = ν_0_, *u_l_* and *v_l_* are real numbers, and *u*_−*l*_ = *u_l_* and *v*_−*l*_ = −*v_l_* are even- and odd-parity components, respectively. To ensure a real function ν_p_, we have ν_*l*_ = (*u_l_* − *iv_l_*)/2 and $\nu _{-l}=\nu _{l}^{\ast }$. According to the reflection symmetry, ν_p_ can be further written as the sum of the symmetric part $\nu _{\mathrm{p}}^{+}$ and the anti-symmetric part $\nu _{\mathrm{p}}^{-}$:


(10a) \begin{eqnarray*} \nu _{\mathrm{p}} = \nu _{\mathrm{p}}^{+} + \nu _{\mathrm{p}}^{-}, \end{eqnarray*}

(10b) \begin{eqnarray*} \nu _{\mathrm{p}}^{+} = \nu _{0}+\sum _{l=1}^{\infty }u_{l}\cos {}(l\theta _{\mathrm{p}}), \end{eqnarray*}

(10c) \begin{eqnarray*} \nu _{\mathrm{p}}^{-} = \sum _{l=1}^{\infty }v_{l}\sin {}(l\theta _{\mathrm{p}}). \end{eqnarray*}

**Figure 2.**
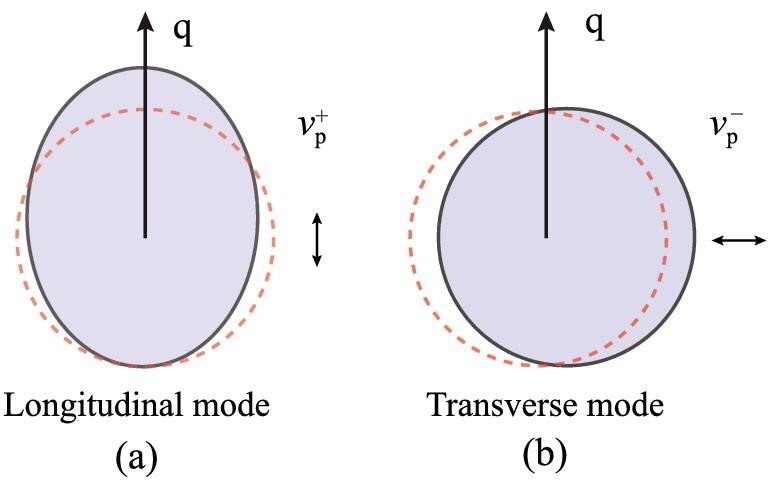
Collective modes in two dimensions. The collective mode is propagating along the wave vector q. Under the reflection about q, i.e., θ → − θ, the longitudinal mode $\nu _{\mathrm{p}}^{+}$ (a) is symmetric, while the transverse mode $\nu _{\mathrm{p}}^{-}$ (b) is anti-symmetric.

As illustrated in Fig. 2, the fluctuations $\nu _{\mathrm{p}}^{+}$ and $\nu _{\mathrm{p}}^{-}$ describe the coherent motion (and/or deformation) of the Fermi surface along and perpendicular to the propagation wave vector q, respectively. Therefore, there are only two kinds of collective modes in two dimensions: the longitudinal modes $\nu _{\mathrm{p}}^{+}$ and the transverse modes $\nu _{\mathrm{p}}^{-}$.

### Two incompressibility conditions

First of all, the simplest and the most important collective mode is the compression and expansion mode, which is characterized by the component $\nu _{0}^{0}$ in three dimensions and the component ν_0_ in two dimensions. Then an incompressible electronic liquid will be achieved if and only if $\nu _{0}^{0}=0$ or ν_0_ = 0 for any wave vector q and frequency ω.

With the help of Landau’s kinetic equation, we have found two incompressibility conditions as follows:

-
$1+{F_{1}^{s}}/{d}=0$ , which gives rise to a gapless QSL state that is a charge insulator and a thermal conductor;
-
$F_{0}^{s}\rightarrow +\infty$ , which leads to a conventional insulator that is both charge and thermal insulating.

We noted that the two incompressibility conditions are rigorous without any further assumption.

### Zero sounds in three dimensions

Zero sound is the density fluctuation in the collisionless regime, ωτ ≫ 1, where the quasiparticles no longer have time to relax to equilibrium in one period of the sound. Thus, the liquid no longer remains in local thermodynamic equilibrium; the character of the sound propagation will be dominated by the quasiparticle interactions.

In the collisionless regime, we can safely neglect the collision integral $I[n_{\mathrm{p}^{\prime }}]$ in (5), resulting in


(11) \begin{eqnarray*} \frac{\partial \delta n_{\mathrm{p}}}{\partial t}+\vec{v}_{\mathrm{p}}\cdot \nabla _{\mathrm{r}}\bigg ( \delta n_{\mathrm{p}}-\frac{\partial n_{\mathrm{p}}^{0}}{\partial \varepsilon _{\mathrm{p}}}\delta \varepsilon _{\mathrm{p} }\bigg ) =0. \end{eqnarray*}

The variation of local energy comes from the applied scalar field *U*_p_ as well as the quasiparticle interaction,


\begin{eqnarray*} \delta \varepsilon _{\mathrm{p}}(\mathrm{r},t)=U_{\mathrm{p}}(\mathrm{r},t)+\sum _{\mathrm{p}^{\prime }}f_{\mathrm{pp}^{\prime }}^{s}\delta n_{\mathrm{p}^{\prime }}. \end{eqnarray*}

To excite a sound mode with an angular momentum *m*, the applied scalar field takes the form $U_{\mathrm{p}}( \mathrm{r},t) =Ue^{im\phi _{\mathrm{p}}}e^{i(\mathrm{q}\cdot \mathrm{r}-\omega t)}$. Substituting this into (11) leads to


(12) \begin{eqnarray*} \nu _{\mathrm{p}}+\frac{\mathrm{q}\cdot \vec{v}_{\mathrm{p}}}{\omega - \mathrm{q}\cdot \vec{v}_{\mathrm{p}}}\sum _{\mathrm{p}^{\prime }}f_{\mathrm{pp}^{\prime }}^{s}\frac{\partial n_{\mathrm{p}^{\prime }}^{0}}{\partial \varepsilon _{\mathrm{p}^{\prime }}}\nu _{\mathrm{p}^{\prime }}=\frac{(\mathrm{q}\cdot \vec{v}_{\mathrm{p}})U{}e^{im\phi _{\mathrm{p}}}}{\omega -\mathrm{q}\cdot \vec{v}_{\mathrm{p}}}\!.\!\!\!\!\!\!\!\! \\ \end{eqnarray*}

#### Algebraic equations

From (12), we found that Landau’s kinetic equation can be expressed in terms of $\nu _{l}^{m}$, and two sets of algebraic equations follow, i.e.,


(13a) \begin{eqnarray*} \frac{\nu _{l}^{m}}{2l+1}+\sum _{l^{\prime }=m}^{\infty }F_{l^{\prime }}^{s}\Omega _{ll^{\prime }}^{m}(s) \frac{\nu _{l^{\prime }}^{m}}{2l^{\prime }+1}=-\Theta _{l}^{m}(s)U,\\ \end{eqnarray*}

which is referred to as ‘type I’ hereafter, and


(13b) \begin{eqnarray*} \nu _{l}^{m}s &&\!\! -\nu _{l-1}^{m}\bigg ( 1+\frac{F_{l-1}^{s}}{2l-1}\bigg ) \frac{\sqrt{l^{2}-m^{2}}}{2l-1} \\ &&-\, \nu _{l+1}^{m} \bigg( 1+\frac{F_{l+1}^{s}}{2l+3}\bigg) \frac{\sqrt{(l+1)^{2}-m^{2}}}{2l+3} \\ &&\qquad =\alpha _{l}^{m}U, \end{eqnarray*}

which is referred to as ‘type II’. Here,


(14) \begin{eqnarray*} s\equiv \frac{\omega }{qv_{F}}\quad \end{eqnarray*}

is a dimensionless parameter, *q* = |q| and *v_F_* is the Fermi velocity. We define $\Omega _{ll^{\prime }}^{m}(s)$, $\Theta _{l}^{m}(s)$, and $\alpha _{l}^{m}$ in the Method section, and the first few nonzero $\Omega _{ll^{\prime }}^{m}$ and $\Theta _{l}^{m}$ can be found in Table 2 in the Method section.

**Table 1.** A summary for sound modes in *d* = 2, 3. L, longitudinal mode; T, transverse mode; (i), $1+{F_{1}^{s}}/{d}=0$ (QSL condition); (ii), $F_{0}^{s}\rightarrow +\infty$ (conventional insulator).

	Mode	*d* = 2	*d* = 3
		Minimal model: three-channel model ($F_{0}^{s},F_{1}^{s},F_{2}^{s}$)	
Zero sound	L	Variable: *u*_0_, *u*_1_, *u*_2_	Variable: ν_0_, ν_1_, ν_2_
		(i) or (ii), & $F_{2}^{s}>2 \Rightarrow s\simeq 1+\frac{1}{2}\left(\frac{F_{2}^{s}-2}{3F_{2}^{s}-2}\right)^2$	(i) or (ii), & $F_{2}^{s}>\frac{10}{3} \Rightarrow s\simeq 1+2\, \text{exp}^ {\big\lbrace -\frac{11}{3}-\frac{50}{9}\frac{1}{F_{2}^{s}-10/3} \big\rbrace} $
		(i) & $F_{2}^{s}\rightarrow \infty \Rightarrow {}s\simeq \sqrt{\frac{F_{2}^{s}}{8}-\frac{3}{4}}$	(i) & $F_{2}^{s}\rightarrow \infty \Rightarrow {}s\simeq \frac{3}{5}\sqrt{\frac{F_{2}^{s}}{7}-\frac{5}{3}}$
		(ii) & $2<F_{2}^{s}\ll {}F_{0}^{s} \Rightarrow {}s\simeq \sqrt{\frac{F_{0}^{s}}{2}\left(1+\frac{F_{1}^{s}}{2}\right)}$	(ii) & $\frac{10}{3}<F_{2}^{s}\ll {}F_{0}^{s} \Rightarrow s\simeq \sqrt{\frac{F_{0}^{s}}{3}\left(1+\frac{F_{1}^{s}}{3}\right)}$
		Minimal model: two-channel model ($F_{0}^{s},F_{1}^{s}$)	
	T	Variable: *v*_0_, *v*_1_	Variable: ν_0_, ν_1_
		(i) & $0<F_{2}^{s}<\frac{2}{3}\Rightarrow s\simeq 1+\frac{1}{8}\left(\frac{3F_{2}^{s}-2}{F_{2}^{s}-2}\right)^{2}$	(i) & $F_{2}^{s}\rightarrow \frac{15}{2}+0^{+}\Rightarrow {}s\rightarrow {}1+0^{+}$
		Minimal model: two-channel model ($F_{m}^{s},F_{m+1}^{s}$)	
	*m* ≥ 2	None	Variable: $\nu _{m}^{m},\nu _{m+1}^{m}$
			$1+\frac{F_{m}^{s}}{2m+1}\rightarrow 0^{+}$ & $\frac{2F_{m+1}^{s}}{(2m+1)(2m+3)}\rightarrow 1+0^{+}\Rightarrow s\rightarrow 1+0^{+}$
		Minimal model: two-channel model ($F_{0}^{s},F_{1}^{s}$)	
First sound	L	Variable: *u*_0_, *u*_1_	Variable: ν_0_, ν_1_
		$c_{1}=v_{F}\sqrt{\frac{1}{d}\left(1+F_{0}^{s}\right)\left(1 +\frac{F_{1}^{s}}{d}\right)}$	

**Table 2.** The functions used in algebraic equations in *d* = 2, 3, and the relations among them. Here L denotes the longitudinal mode and T denotes the transverse mode. Note that, for the physical situation, we always choose *s* with positive imaginary part (can be infinitesimal). For instance, for a real value of *s*, Ω_00_(*s*) = 1 − (*s*/2)ln |(*s* + 1)/(*s* − 1)| + *i*π*s*θ(1 − |*s*|)/2.

*d* = 2, L	*d* = 2, T	*d* = 3, *m* = 0	*d* = 3, *m* ≥ 0
$\Pi _{ll^{\prime }}=\Pi _{l^{\prime }l}$	$\Xi _{ll^{\prime }}=\Xi _{l^{\prime }l}$	$\Omega _{ll^{\prime }}:=\Omega _{ll^{\prime }}^{0}$ , $\Omega _{ll^{\prime }}^{m}=\Omega _{l^{\prime }l}^{m}$	
$\Pi _{l1}-s\Pi _{l0}=\frac{1}{2}\delta _{l1}$	$\Xi _{l2}-2s\Xi _{l1}=\frac{1}{2}\delta _{l2}$	$\Omega _{l,m+1}^{m}-s\sqrt{2m+1}\Omega _{lm}^{m}=\frac{1}{2m+3}\delta _{l,m+1}$	
	Ξ_*l*0_ = 0	$\Omega _{m+1,m+1}^{m+1}=\frac{2m+1}{2m+2}\left(1-s^{2}\right)\Omega _{mm}^{m}-\frac{1}{\left(2m+2\right)\left(2m+3\right)}$	
$\Pi _{00}=1-\frac{s}{\sqrt{s^{2}-1}}$	$\Xi _{11} =\frac{1-2s^{2}}{2}\Pi _{00}$	$\Omega _{00}=1-\frac{s}{2}\ln \frac{s+1}{s-1}$	$\Omega _{00}^{0}=1-\frac{s}{2}\ln \frac{s+1}{s-1}=\Omega _{00}$
Π_10_ = *s*Π_00_	Ξ_21_ = 2*s*Ξ_11_	Ω_10_ = *s*Ω_00_	$\Omega _{11}^{1}=\frac{1}{2}(1-s^{2})\Omega _{00}^{0}-\frac{1}{6}$
$\Pi _{11}=s\Pi _{10}+\frac{1}{2}$	$\Xi _{22}=2s\Xi _{21}+\frac{1}{2}$	$\Omega _{11}=s\Omega _{10}+\frac{1}{3}$	$\Omega _{22}^{2}=\frac{3}{4}(1-s^{2})\Omega _{11}^{1}-\frac{1}{20}$
Π_20_ = 1 + (2*s*^2^ − 1)Π_00_		$\Omega _{20}=\frac{1}{2}+\frac{1}{2}(3s^{2}-1)\Omega _{00}$	
Π_21_ = *s*Π_20_		Ω_21_ = *s*Ω_20_	
$\Pi _{22}=\frac{1}{2}+(2s^{2}-1)\Pi _{20}$		$\Omega _{22}=\frac{1}{5}+\frac{1}{2}(3s^{2}-1)\Omega _{20}$	

It is easy to verify that if $\nu _{l}^{m}$ is a solution to (13) then $\bar{\nu }_{l}^{m}=(-1)^{m}\nu _{l}^{-m}$ gives rise to another solution to both type-I and type-II algebraic equations. We therefore focus on *m* ≥ 0 unless otherwise specified.

The type-I equation (13a) consists of an infinite set of equations, and can be derived from the type-II equation (13b) directly and vice versa. Both rigorous and approximate results can be obtained from these algebraic equations, as will be seen. For instance, the two incompressibility conditions can be obtained from type-II algebraic equations without any further approximation. Moreover, one can solve (13a) by introducing a truncation for $F_{l}^{s}$, namely, keep a finite number of $F_{l}^{s}$ and neglect others. Then possible collective modes describing oscillations of the system can be found from the poles of the response function ν_*l*_/*U*, which depends on variable *s* only.

*Minimal model.* As mentioned above, a physical truncation for $F_{l}^{s}$ can be utilized to solve equations (13). By such a truncation, a finite number (*n*) of $\nu _{l}^{m}$ form a closed set of algebraic equations, which gives rise to an ‘*n*-channel model’. To find a non-trivial solution for a specific collective mode, a minimal *n* is required, and the corresponding *n*-channel model is called a ‘minimal model’.

#### Longitudinal mode

We begin with some rigorous results for *m* = 0 modes. Let us examine the first equation of type II, say, the one with *l* = *m* = 0 in (13b). Note that the second term on the left-hand side vanishes automatically, and we have


(15) \begin{eqnarray*} \nu _{0}s-\bigg ( 1+\frac{F_{1}^{s}}{3}\bigg )\frac{\nu _{1}}{3}=0. \end{eqnarray*}

This leads to incompressibility condition (i), $1+{F_{1}^{s}}/{d}=0$ with *d* = 3, which is the exact QSL condition in (4c).

On the other hand, the second one with *m* = 0 and *l* = 1 in (13b) reads


(16) \begin{eqnarray*} \nu _{1}s-(1+F_{0}^{s})\nu _{0}-\bigg ( 1+\frac{F_{2}^{s}}{5}\bigg ) \frac{2}{5}\nu _{2}=U.\\ \end{eqnarray*}

This leads to incompressibility condition (ii): $F_{0}^{s}\rightarrow +\infty$. This is nothing but the strong repulsion limit in which a metal-to-conventional-insulator Mott transition occurs.

In addition to the rigorous results for incompressibility, longitudinal zero sound modes can be studied via a minimal model, which is a three-channel model by the following truncation: keep $F_{0}^{s}$, $F_{1}^{s}$ and $F_{2}^{s}$, and set $F_{l}^{s}=0$ for *l* ≥ 3. With such a truncation, the type-I algebraic equation (13a) consisting of a series of algebraic equations can be classified into two categories: (i) *l* ≥ 3 and (ii) *l* = 0, 1, 2. The algebraic equations in the second category form a closed set that are linear equations of the variables ν_0_, ν_1_ and ν_2_. Then the mode frequency is determined by the secular equation


(17) \begin{eqnarray*} && \bigg [F_{0}^{s}\bigg (1+\frac{F_{1}^{s}}{3}\bigg )+F_{1}^{s}s^{2}\bigg ] \bigg [1+F_{2}^{s}\bigg (\Omega _{22}-\frac{\Omega _{20}^{2}}{\Omega _{00}}\bigg )\bigg ] \Omega _{00} \\ &&\quad +\bigg (1+\frac{F_{1}^{s}}{3}\bigg )(1+F_{2}^{s}\Omega _{22}) =0, \end{eqnarray*}

where $\Omega _{ll^{\prime }}$ is defined as $\Omega _{ll^{\prime }}:=\Omega _{ll^{\prime }}^{0}$. A sufficient condition for a real solution *s*(> 1) to (17) is found to be


(18) \begin{eqnarray*} &&\bigg [9+\bigg (1+\frac{F_{1}^{s}}{3}\bigg )(1-3F_{0}^{s})\bigg ]\frac{F_{2}^{s}}{10} \\ && +\, \bigg (1+\frac{F_{1}^{s}}{3}\bigg )(3+F_{0}^{s})-3 >0. \end{eqnarray*}

(When *s* > 1, the sound speed exceeds the Fermi velocity *v_F_*, so that the collective mode will survive the Landau damping.) Note that, under either incompressibility condition (i) or (ii), the above inequality reduces to $F_{2}^{s}>10/3$. [In the limit of $F_{2}^{s}\rightarrow {}0$ and $1+F_{1}^{s}/3\rightarrow {}0$, the inequality in (18) cannot be satisfied. Therefore a two-channel model consisting of $F_{0}^{s}$ and $F_{1}^{s}$ (thereby ν_0_ and ν_1_) only does not host a weakly damped *m* = 0 zero sound mode under the incompressibility condition $1+F_{1}^{s}/d=0$, and the three-channel model is a minimal model for studying the longitudinal zero sound mode in a gapless QSL.]

In general, a solution *s* to (17) must be found numerically. However, approximately analytical solutions can be found in two limits, *s* → 1 + 0^+^ and *s* → ∞, as long as the sufficient condition (18) is satisfied. In particular, in two of the incompressible limits,

1. for either (i) $1+{F_1^s}/{3}=0$ or (ii) $F_{0}^{s}\rightarrow +\infty$, there exists a weakly damped longitudinal zero sound mode with
  (19a) \begin{eqnarray*} s\simeq 1+2 \, \text{exp}\bigg \lbrace -\!\frac{11}{3}-\frac{50}{9}\frac{1}{F_{2}^{s}-10/3}\bigg \rbrace ,\\ \end{eqnarray*}
  as long as $F_{2}^{s}>10/3$; note that this solution depends only on $F_{2}^{s}$ but neither $F_{0}^{s}$ nor $F_{1}^{s}$;
2. when (i) $1+{F_1^s}/{3}=0$ and $F_{2}^{s}\rightarrow +\infty$, an additional weakly damped longitudinal zero sound mode will occur with
  (19b) \begin{eqnarray*} s\simeq \frac{3}{5}\sqrt{\frac{F_{2}^{s}}{7}-\frac{5}{3}}; \end{eqnarray*}
3. when (ii) $F_{0}^{s}\rightarrow +\infty$ and $10/3<F_{2}^{s}\ll {}F_{0}^{s}$, an extra solution to (17) is allowed,
  (19c) \begin{eqnarray*} s\simeq \sqrt{\frac{F_{0}^{s}}{3}\bigg (1+\frac{F_{1}^{s}}{3}\bigg )}, \end{eqnarray*}
  which takes the same form as that of first sound if $1+{F_{1}^{s}}/{3}>0$ and $F_{0}^{s}\rightarrow +\infty$, as will been seen later.

#### The *m* ≥ 1 zero sound modes

*Minimal models.* To study generic *m* ≥ 1 modes, we keep the Landau parameters up to $F_{m+1}^{s}$, such that there allows (*m* + 2)-finite $F_{l}^{s}(l=0,\dots ,m+1)$, and $F_{l}^{s}=0$ for *l* > *m* + 2. An interesting observation on equations (13) is the following. The Landau parameter $F_{l}^{s}$ does not affect an *m* > *l* sound mode. For instance, $F_{0}^{s}$ does not affect the transverse (*m* = 1) mode. Thus, for a fixed *m* > 0, there are only two relevant Landau parameters, $F_{m}^{s}$ and $F_{m+1}^{s}$, that are active in equations (13). The truncation of these (*m* + 2)-Landau parameters will result in a two-channel model consisting of two variables, $\nu _{m}^{m}$ and $\nu _{m+1}^{m}$, only.

Such a two-channel model is a minimal model for studying *m* ≥ 1 zero sound modes, and can be derived from equations (13). The mode frequency is determined by the secular equation


(20) \begin{eqnarray*} h(s)\!\! &=& [1+F_{m}^{s}\Omega _{mm}^{m}(s)]\bigg (1+\frac{F_{m+1}^{s}}{2m+3}\bigg ) \\ && +\, (2m+1)F_{m+1}^{s}s^2\Omega _{mm}^{m}(s) =0.\\ \end{eqnarray*}

The sufficient condition of a real solution *s*(>1) for *h*(*s*) = 0 is


(21) \begin{eqnarray*} F_{m+1}^{s}>(2m+3)\frac{2m+1-[1+{F_{m}^{s}}/{(2m+1)}]}{2+[1+{F_{m}^{s}}/{(2m+1)}]}.\!\!\!\!\! \\ \end{eqnarray*}

In the limit of $1+{F_{m}^{s}}/{(2m+1)}=0$, the above inequality becomes


(22) \begin{eqnarray*} F_{m+1}^{s}>\frac{(2m+1)(2m+3)}{2}, \end{eqnarray*}

and when $F_{m+1}^{s} = {(2m+1)(2m+3)}/{2}$, there is an exact solution, *s* = 1.

**Figure 3.**
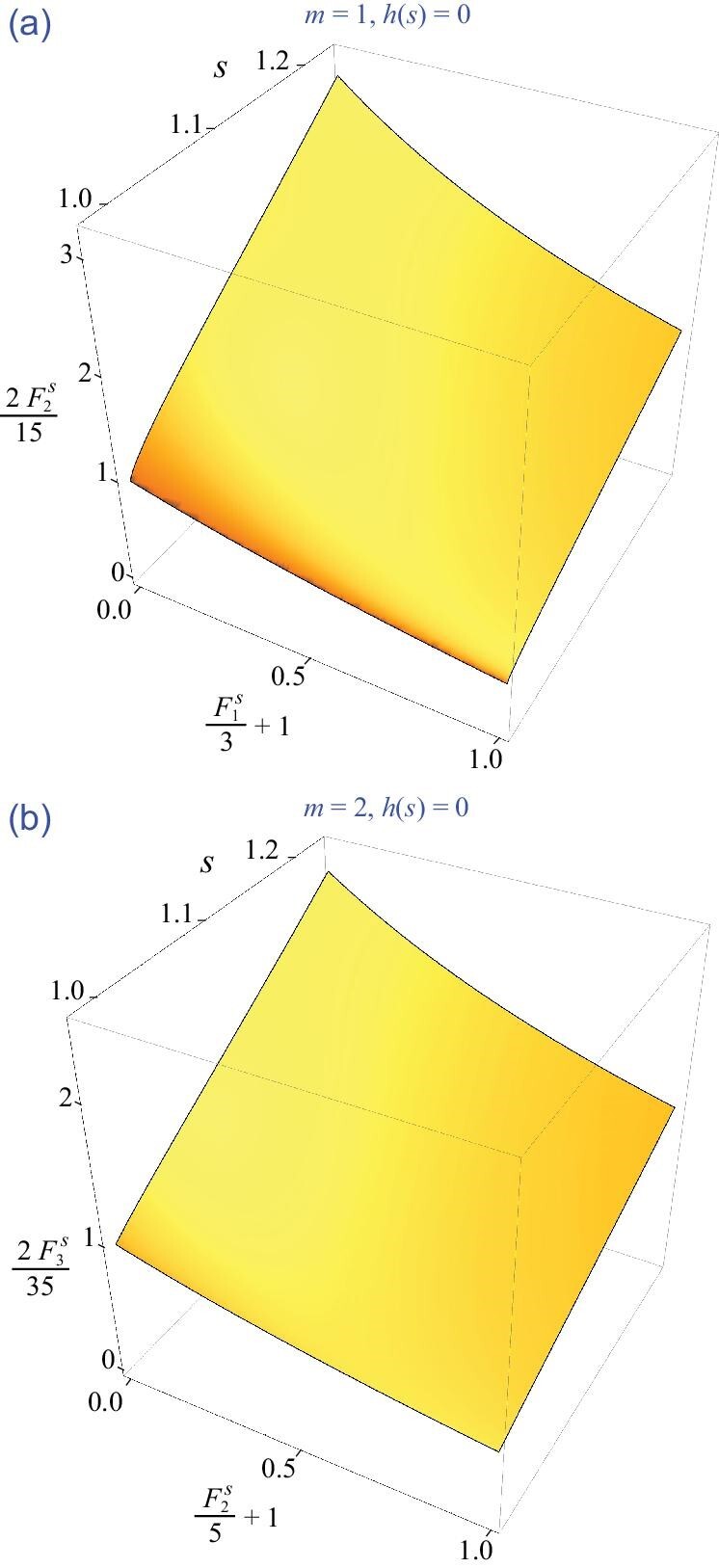
Numerical solutions for *m* ≥ 1 zero sound modes in three dimensions. (a) Transverse (*m* = 1) and (b) quadrupolar (*m* = 2) modes.

In particular, we are interested in transverse (*m* = 1) and quadrupolar (*m* = 2) modes. For these modes, we numerically found that (57) is also a necessary condition for a real solution *s*( > 1) to *h*(*s*) = 0. The numerical solutions have been demonstrated in Fig. 3 as a three-dimensional contour plot in the parameter space $[s,1+{F_{m}^{s}}/{(2m+1)},{2F_{m+1}^{s}}/{(2m+1)(2m+3)}]$. Note that these numerical solutions are all around *s* → 1 + 0^+^.

Indeed, another solution can be found analytically in the large-*s* limit as


(23) \begin{eqnarray*} s\simeq {}\sqrt{\frac{1}{2m+3}\bigg [\frac{F_{m}^{s}}{2m+1}\bigg (1+\frac{F_{m+1}^{s}}{2m+3}\bigg ) +\frac{3F_{m+1}^{s}}{2m+5}\bigg ]}\!\!\!\!\!\!\!\! \\ \end{eqnarray*}

for arbitrary *m* ≥ 1, as long as the condition


(24) \begin{eqnarray*} \frac{F_{m}^{s}}{2m+1}\bigg (1+\frac{F_{m+1}^{s}}{2m+3}\bigg )+\frac{3F_{m+1}^{s}}{2m+5}\gg 2m+3\\ \end{eqnarray*}

is satisfied.

#### Suppression of |*m*| = *l* ≥ 1 modes

Similar to incompressibility conditions (i) $1+{F_{1}^{s}}/{d}=0$ and (ii) $F_{0}^{s}\rightarrow +\infty$ from the suppression of the $\nu _{0}^{0}$ component, we do have other rigorous conditions for suppressing some |*m*| ≥ 1 components. Indeed, when |*m*| = *l* ≥ 1, (13b) gives rise to


(25) \begin{eqnarray*} \!\!\!\!\!\!\! \nu _{l}^{\pm {}l}s-\nu _{l+1}^{\pm {}l}\bigg ( 1+\frac{F_{l+1}^{s}}{2l+3}\bigg ) \frac{\sqrt{2l+1}}{2l+3}=0. \end{eqnarray*}

Therefore, when

\begin{eqnarray*} 1+\frac{F_{l+1}^{s}}{2l+3}=0, (26) \end{eqnarray*}

the ${m}$ = ±${l}$ components will be completely suppressed in low energies, i.e., $\nu _{l}^{m=\pm {}l}=0$, and thereby acquire an excitation energy gap.

*Nematic QSL state.* It is remarkable that the suppression of transverse current (*l* = 1 and *m* = ±1) modes will give rise to a ‘nematic’ QSL state that cannot be described by the usual *U*(1) gauge theory, in which the transverse current mode is gapless. Such a nematic QSL is a Mott insulator, whose spinon Fermi surface undergoes a nematic transition because of the Pomeranchuk instability, breaks the rotational symmetry spontaneously and has an elliptical shape. It was suggested that a Pomeranchuk instability may occur in *l* ≥ 2 [or *l* ≥ 1 in (26)] channels [19], while the gauge invariance and charge (spin) conservation law prevent the Pomeranchuk instability in *l* = 1 channels [16,20,21]. Therefore, the nematic QSL state is allowed. Moreover, the *l* = 1 and *m* = 0 modes will become strongly damping under these conditions [see equations (19)]. Therefore, all the dipolar (*l* = 1) modes vanish in low energy. However, quadrupolar and higher angular momentum modes, i.e., *l* ≥ 2 modes, can still be gapless, even though *l* = 1 modes are gapped or damping strongly.

*Hierarchy of QSL states.* The possibility of the suppression of |*m*| = *l* modes and the inequality in (22) suggest that there is a hierarchy structure for gapless QSL states:

when

\begin{eqnarray*} && 1+\frac{F_{l}^{s}}{2l+1}\left\lbrace \begin{array}{@{}l@{\quad }l@{}}\, >0, & l=0 \text{ or } l\ge n+2,\\ \, =0, & l=1,\dots ,n,\\ \, >\displaystyle\frac{2l+1}{2}, & l=n+1, \end{array}\right. (27) \end{eqnarray*}

the Landau-type effective theory defined in (1) describes a gapless QSL state in three dimensions, on which |${m}$| = 0, …, ${n}$ zero sound modes are gapped, while |${m}$| = ${n}$ + 1 zero sound modes are gapless. Here ${n}$ is a positive integer, and ${m}$ is the ‘magnetic’ quantum number along the collective mode propagating direction $\hat{\mathrm{q}}$.

Such a hierarchy structure indicates that a QSL may possess a more complicate spinon Fermi surface than an ellipsoid in the nematic QSL, while they are all topologically identical to a sphere.

### First sound in three dimensions

First sound is the ordinary sound in a liquid, i.e., density fluctuations in the hydrodynamic regime ωτ ≪ 1. In this regime, the collision integral $I[n_{\mathrm{p}^{\prime }}]\sim \delta n /\tau$ becomes significant, and the system is essentially in local thermodynamic equilibrium. In such a strong collision regime, following Abrikosov and Khalatnikov [22], we assume that the collision integral takes the form


(28a) \begin{eqnarray*} I[n_{\mathrm{p}}] = - \frac{1}{\tau _\mathrm{p}}[\delta {}n_{\mathrm{p}}-\langle \delta {}n_{\mathrm{p}} \rangle -3\langle \delta {}n_{\mathrm{p}}\cos \theta _{\mathrm{p}}\rangle \cos \theta _{\mathrm{p}}]\!\!\!\!\!\! \\ \end{eqnarray*}

and that


(28b) \begin{eqnarray*} \frac{1}{\tau _\mathrm{p}} = \frac{1}{\tau _{i}} + \frac{(\varepsilon _{\mathrm{p}}-\varepsilon _{F})^{2}}{\varepsilon _{a}}+\frac{(k_{\beta }T)^{2}}{\varepsilon _{b}}, \end{eqnarray*}

where 〈·〉 denotes the average over the solid angle, τ_p_ is the relaxation time and its inverse gives rise to the scattering rate. Here 1/τ_*i*_ is the impurity scattering rate, and the other parts correspond to relaxations due to quasiparticle interactions and thermal fluctuations. The ϵ_*a*_ and ϵ_*b*_ are two characteristic energy scales for quasiparticle interactions and thermal fluctuations, respectively.

With the help of (7), the expectation values in the collision integral *I*[*n*_p_] in (28) can be computed as


(29) \begin{eqnarray*} \langle {}\delta {}n_{\mathrm{p}}\rangle =-\frac{\partial n_{\mathrm{p}}^{0}}{\partial \varepsilon _{\mathrm{p}}}\nu _{0}, \qquad 3\langle {}\delta {}n_{\mathrm{p}}\cos {\theta _{\mathrm{p}}}\rangle =-\frac{\partial n_{\mathrm{p}}^{0}}{\partial \varepsilon _{\mathrm{p}}}\nu _{1}.\\ \end{eqnarray*}

In the presence of an external field like that assumed in the collisionless regime, $U_{\mathrm{p}}( \mathrm{r},t) =Ue^{im\phi _{\mathrm{p}}}e^{i(\mathrm{q}\cdot \mathrm{r}-\omega t)}$, Landau’s kinetic equation can be rewritten in frequency-momentum space as


(30) \begin{eqnarray*} &&(\omega -\mathrm{q}\cdot \vec{v}_{\mathrm{p}})\delta n_{\mathrm{p}} \\ &&+\frac{\partial {}n_{\mathrm{p}}^{0}}{\partial \varepsilon _{\mathrm{p}}}(\mathrm{q}\cdot \vec{v}_{\mathrm{p}})\bigg ( U{}e^{im\phi _{\mathrm{p}}}+\sum _{\mathrm{p}^{\prime }}f_{\mathrm{pp}^{\prime }}^{s}\delta n_{\mathrm{p}^{\prime }}\bigg ) \\ &&\qquad =iI[n_{\mathrm{p}}]. \end{eqnarray*}

In the hydrodynamic limit, *I*[*n*_p_] ∝ 1/τ_p_, and ℏ/τ_p_ is the largest energy scale and does not depend on the azimuthal angle φ_p_ and the corresponding quantum number *m*. Therefore, *m* ≠ 0 modes will damp strongly, and we study the longitudinal (*m* = 0) mode only in this limit.

Substituting (7) into (30), we obtain the type-I algebraic equation in the *U* → 0 limit:


(31) \begin{eqnarray*} &&\frac{\nu _{l}}{2l+1} + \sum \limits_{l^{\prime }}F_{l^{\prime }}^{s}\Omega _{ll^{\prime }}(\tilde{s})\frac{\nu _{l^{\prime }}}{2l^{\prime }+1} \\ &&\qquad = -i\kappa [\Omega _{l0}(\tilde{s}){\nu }_{1}+\Omega _{l}(\tilde{s}){\nu }_{0}] \end{eqnarray*}

with


(32) \begin{eqnarray*} \kappa &=&\frac{1}{\tau {}q{}v_{F}}=\frac{s}{\omega \tau }, \qquad \tilde{s}= \kappa (\omega \tau + i)\\ &=& s\bigg (1+\frac{i}{\omega \tau }\bigg ) \end{eqnarray*}

and


(33) \begin{eqnarray*} \Omega _{l}(\tilde{s})=\frac{1}{2}\int _{-1}^{1}d\mu P_{l}(\mu )\frac{1}{\mu -\tilde{s}}. \end{eqnarray*}

Here the assumption that τ_p_ = τ has been used, and it is easy to verify that $\Omega _{l}(\tilde{s})=({1}/{\tilde{s}})[\Omega _{l0}(\tilde{s})-\delta _{l,0}]$. In comparison with (13a) in the collisionless regime, there appear additional κ terms on the right-hand side of (31) that are induced by collisions.

#### First sound speed

To study the first sound mode, we consider a two-channel model by keeping $F_{0}^{s}$ and $F_{1}^{s}$ only and setting $F_{l}^{s}=0$ for *l* ≥ 2, which is a minimal model for studying first sound. The mode frequency is determined by


(34) \begin{eqnarray*} &&[1+F^{s}_{0}\Omega _{00}+i\kappa \Omega _{0}]\bigg [ \frac{1}{3}(1+F^{s}_{1}\Omega _{11}) +i\kappa \Omega _{10}\bigg ] \\ && - \bigg (\frac{1}{3}F^{s}_{1}\Omega _{10} + i\kappa \Omega _{00}\bigg )( F^{s}_{0}\Omega _{10}+i\kappa \Omega _{1}) =0. \\ \end{eqnarray*}

For ωτ ≪ 1, we have


(35) \begin{eqnarray*} \kappa ^{-1}\rightarrow 0,\qquad \tilde{s}\rightarrow {}i\infty +0^{+}\quad \text{and}\quad \tilde{s}\kappa ^{-1}\rightarrow i.\!\!\!\!\!\!\! \\ \end{eqnarray*}

The functions $\Omega _{ll^{\prime }}(\tilde{s})$ and $\Omega _{l}(\tilde{s})$ can be expanded around $\tilde{s}=i\infty$ asymptotically. Substituting these expansions into (34) leads to the following dispersion relation between ω and q:


(36) \begin{eqnarray*} \bigg (\frac{\omega }{qv_{F}}\bigg )^{2}=\bigg [ \frac{1}{3} \left(1+F_{0}^{s}\right)-\frac{4}{15}i\omega \tau \bigg ]\bigg (1+\frac{1}{3}F_{1}^{s}\bigg ).\!\!\!\!\!\! \\ \end{eqnarray*}

This is exactly the same as equation (10.9) of [22]. In the limit of ωτ ≪ 1, we obtain the first sound speed


(37) \begin{eqnarray*} c_{1}=\frac{\omega }{q}=v_{F}\sqrt{\frac{1}{3} \left(1+F_{0}^{s}\right)\bigg (1+\frac{1}{3}F_{1}^{s}\bigg )}.\\ \end{eqnarray*}

We emphasize that the first sound speed also behaves quite differently under the two incompressibility conditions:

(i) when $1+{F_{1}^{s}}/{3}\rightarrow 0$, *c*_1_ → 0; (ii) when $F_{0}^{s}\rightarrow +\infty$, *c*_1_ → ∞.

### Sound modes in two dimensions

In parallel with three dimensions, we study collective modes in two dimensions. By the reflection symmetry, the longitudinal mode $\nu _{\mathrm{p}}^{+}$ and the transverse mode $\nu _{\mathrm{p}}^{-}$ can be excited by the external scalar fields


(38a) \begin{eqnarray*} U_{+}(\mathrm{r},t)=U{}e^{i(\mathrm{q}\cdot \mathrm{r}-\omega {}t)} \end{eqnarray*}

and


(38b) \begin{eqnarray*} U_{-}(\mathrm{r},t)=[(\hat{\mathrm{n}}\times \hat{\mathrm{q}})\cdot \hat{v}_\mathrm{p}]{}U{}e^{i(\mathrm{q}\cdot \mathrm{r}-\omega {}t)}, \end{eqnarray*}

respectively. Here $\hat{\mathrm{n}}$ is the unit vector normal to the two-dimensional plane and $(\hat{\mathrm{n}}\times \hat{\mathrm{q}})\cdot \hat{v}_\mathrm{p}=\sin \theta _{\mathrm{p}}$. Below we study longitudinal and transverse modes in both collisionless (ωτ ≫ 1) and hydrodynamic (ωτ ≪ 1) regimes.

#### Zero sound in two dimensions

Two types of algebraic equation for zero sound can be derived from Landau’s kinetic equation, as in three dimensions. First, type-I algebraic equations for longitudinal and transverse modes have been derived as


(39) \begin{eqnarray*} \frac{\tilde{u}_{l}}{2}&&+\sum _{l^{\prime }=0}^{\infty }F_{l^{\prime }}^{s}\Pi _{ll^{\prime }}(s) \frac{\tilde{u}_{l^{\prime }}}{2} =-\Pi _{l0}(s)U, \\ \frac{v_{l}}{2}&&+\sum _{l^{\prime }=0}^{\infty }F_{l^{\prime }}^{s}\Xi _{ll^{\prime }}(s) \frac{v_{l^{\prime }}}{2} =-\Xi _{l1}(s)U, \end{eqnarray*}

where $\tilde{u}_{l}=u_{l}+\delta _{l0}u_{0}$, and $\Pi _{ll^{\prime }}(s)$ and $\Xi _{ll^{\prime }}(s)$ are defined in the Method section. The first few nonzero $\Pi _{ll^{\prime }}$ and $\Xi _{ll^{\prime }}$ can be found in Table 2 in the Method section.

Second, type-II algebraic equations for the longitudinal mode have been found as


(40a) \begin{eqnarray*} u_{l}s &-& \bigg (1+\frac{F_{l-1}^{s}+\delta _{l1}F_{0}^{s}}{2}\bigg ) \frac{u_{l-1}+\delta _{l1}u_{0}}{2} \\ &-&\, \bigg (1+\frac{F_{l+1}^{s}}{2}\bigg )\frac{u_{l+1}}{2} =\delta _{l1}U, \end{eqnarray*}

where *l* = 0, 1, 2, … and *u*_−1_ = 0 has been set for convention; type-II algebraic equations for the transverse mode read


(40b) \begin{eqnarray*} v_{l}s &-&\bigg (1+\frac{F_{l-1}^{s}}{2}\bigg )\frac{v_{l-1}}{2}\\ &-& \bigg (1+\frac{F_{l+1}^{s}}{2}\bigg )\frac{v_{l+1}}{2}=\delta _{l2}\frac{U}{2},\\ \end{eqnarray*}

where *l* = 1, 2, 3, … and *v*_0_ = 0 has been set for convention.

*Incompressibility conditions in two dimensions.* Similarly as in three dimensions, the first two components in type-II algebraic equation (40a) read


(41) \begin{eqnarray*} u_{0}s &-& \bigg (1+\frac{F_{1}^{s}}{2}\bigg )\frac{u_{1}}{2}=0, \\ u_{1}s &-& (1+F_{0}^{s})u_{0}-\bigg (1+\frac{F_{2}^{s}}{2}\bigg )\frac{u_{2}}{2}=U. \\ \end{eqnarray*}

These lead to two incompressibility conditions in two dimensions: (i) $1+{F_{1}^{s}}/{2}=0$ and (ii) $F_{0}^{s}\rightarrow \infty$.

#### Longitudinal mode $\nu _{\mathrm{p}}^{+}$

As in three dimensions, the minimal model for studying the longitudinal zero sound mode in two dimensions is a three-channel model consisting of three Landau parameters, $F_{0}^{s},F_{1}^{s},F_{2}^{s}$, and three variables, *u*_0_, *u*_1_, *u*_2_, only. The mode frequency is determined by the secular equation


(42) \begin{eqnarray*} &&\bigg [F_{0}^{s}\bigg (1+\frac{F_{1}^{s}}{2}\bigg )+F_{1}^{s}s^{2}\bigg ] \\ &&\qquad \times \bigg [1+F_{2}^{s}\bigg (\Pi _{22}-\frac{\Pi _{20}^{2}}{\Pi _{00}}\bigg )\bigg ]\Pi _{00} \\ &&\qquad \ +\bigg (1+\frac{F_{1}^{s}}{2}\bigg )(1+F_{2}^{s}\Pi _{22}). \end{eqnarray*}

It turns out that the secular equation always has at least one real solution *s*(>1) as long as the inequality


(43) \begin{eqnarray*} &&\bigg [1-\frac{F_{0}^{s}}{2}\bigg (1+\frac{F_{1}^{s}}{2}\bigg )\bigg ]F_{2}^{s}\\ &&\qquad+ \bigg [F_{0}^{s}\bigg (1+\frac{F_{1}^{s}}{2}\bigg )+F_{1}^{s}\bigg ]>0 \end{eqnarray*}

is satisfied.

In the two limits of incompressibility, either (i) $1+{F_1^s}/{2}=0$ or (ii) $F_{0}^{s}\rightarrow +\infty$, the inequality becomes $F_{2}^{s}>2$. Then we have the following approximate solutions to (42) under two incompressibility conditions.

1. For either (i) $1+{F_1^s}/{2}=0$ or (ii) $F_{0}^{s}\rightarrow +\infty$, there exists a solution,
  (44a) \begin{eqnarray*} s\simeq {}1+\frac{1}{2}\bigg (\frac{F_{2}^{s}-2}{3F_{2}^{s}-2}\bigg )^2, \end{eqnarray*}
  as long as $F_{2}^{s}>2$.
2. When (i) $1+{F_1^s}/{2}=0$ and $F_{2}^{s}\rightarrow {}+\infty$, an additional solution *s* → +∞ will appear and reads
  (44b) \begin{eqnarray*} s\simeq \sqrt{\frac{F_{2}^{s}}{8}-\frac{3}{4}}. \end{eqnarray*}
3. When (ii) $F_{0}^{s}\rightarrow +\infty$ and $2<F_{2}^{s}\ll {}F_{0}^{s}$, another solution to (42) is allowed,
  (44c) \begin{eqnarray*} s\simeq \sqrt{\frac{F_{0}^{s}}{2}\bigg (1+\frac{F_{1}^{s}}{2}\bigg )}. \end{eqnarray*}

#### Transverse mode $\nu _{\mathrm{p}}^{-}$

The minimal model for the transverse zero sound mode is a two-channel model, in which we keep only $F_{1}^{s}$ and $F_{2}^{s}$ and let $F_{l}^{s}=0$ for *l* ≥ 3. The mode frequency can be determined by


\begin{eqnarray*} \bar{h}_{2}(s)&\equiv& [1+F_{1}^{s}\Xi _{11}(s)] \bigg (1+\frac{F_{2}^{s}}{2}\bigg )\\ && +\, 4s^{2}F_{2}^{s}\Xi _{11}(s) = 0. \end{eqnarray*}

To have a real solution *s* to $\bar{h}_{2}(s)=0$, one requires


(45) \begin{eqnarray*} F_{2}^{s}\bigg [F_{1}^{s}\bigg (1+\frac{F_{2}^{s}}{2}\bigg )+4F_{2}^{s}\bigg ]<0. \end{eqnarray*}

Under the QSL condition $1+{F_{1}^{s}}/{2}=0$, (45) gives rise to $0<F_{2}^{s}<\frac{2}{3}$, and the approximate solution is simplified as


(46) \begin{eqnarray*} s=1+\frac{1}{8}\bigg (\frac{3F_{2}^{s}-2}{F_{2}^{s}-2}\bigg )^{2}. \end{eqnarray*}

**Figure 4.**
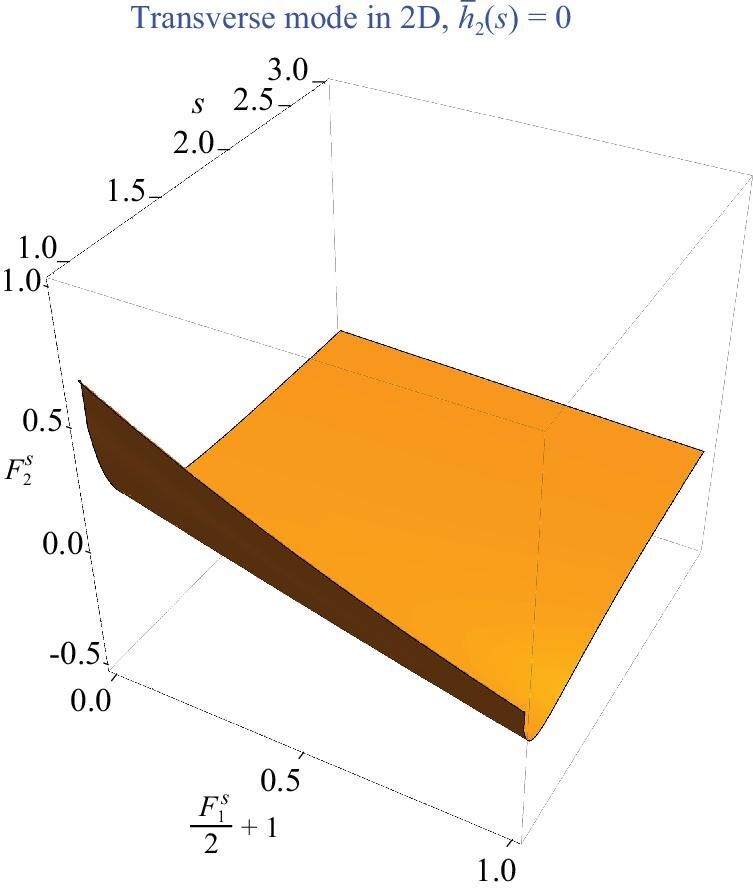
Numerical solutions for the transverse mode in two dimensions.

The numerical solution to (45) can be found in Fig. 4, where the zeros of the function $\bar{h}_{2}(s)$ are plotted as a three-dimensional contour in the parameter space $(s,1+{F_{1}^{s}}/{2},F_{2}^{s})$. In order to have a real solution *s* to $\bar{h}_{2}(s)=0$, the Landau parameter $F_{2}^{s}$ should be restricted to a finite region given in (45). This is quite different from the situation in three dimensions, in which a positive and sufficiently large $F_{2}^{s}$ always gives rise to a weakly damped transverse mode.

#### First sound in two dimensions

The minimal model for studying the first sound mode is the two-channel model with finite $F_{0}^{s}$ and $F_{1}^{s}$ and vanishing $F_{l}^{s}=0$ for *l* ≥ 2. Similar analyses as those in three dimensions lead to the mode frequency equation


(47) \begin{eqnarray*} && (1+F_{0}^{s}\Pi _{00}+i\kappa \Pi _{0})\bigg (\frac{1}{2}(1+F_{1}^{s}\Pi _{11}) +i\kappa \Pi _{10}\! \bigg ) \\ && -\, \bigg (\frac{1}{2}F_{1}^{s}\Pi _{01}+i\kappa \Pi _{00}\bigg )(F_{0}^{s}\Pi _{10}+i\kappa \Pi _{1}) = 0 \\ \end{eqnarray*}

and the dispersion relation


(48) \begin{eqnarray*} \bigg (\frac{\omega }{qv_{F}}\bigg )^{2}&&= \frac{1}{2}(1+F_{0}^{s}) \bigg (1+\frac{F_{1}^{s}}{2}\bigg )\\ &&\quad -\, \frac{1}{4}i\omega \tau \bigg (1+\frac{F_{1}^{s}}{2}\bigg ). \end{eqnarray*}

In the limit of ωτ ≪ 1, the first sound speed reads


(49) \begin{eqnarray*} c_{1}=\frac{\omega }{q}=v_{F}\sqrt{\frac{1}{2}(1+F_{0}^{s}) \bigg (1+\frac{1}{2}F_{1}^{s}\bigg )}.\\ \end{eqnarray*}

## CONCLUSIONS AND DISCUSSIONS

Exploiting a Landau-type effective theory and the Landau’s kinetic equation, we have studied collective modes, i.e., zero and first sound modes, for an electronic liquid in *d* = 2, 3 spatial dimensions.

- All these collective modes can be classified according to symmetries, which include
  - longitudinal and transverse modes in two dimensions;
  - longitudinal, transverse and higher angular momentum (*m* ≥ 2) modes in three dimensions.
- Two types of algebraic equation have been derived from Landau’s kinetic equation, and *rigorous* results for zero sound have been obtained as follows.
  - The condition $1+{F_{1}^{s}}/{d}=0\Rightarrow \nu _{0}=0$, which gives rise to a gapless QSL that is a charge insulator and thermal conductor.
  - The condition $F_{0}^{s}\rightarrow +\infty \Rightarrow \nu _{0}=0$, which leads to a conventional insulator that is both charge and thermal insulating.
  - For *d* = 3, $1+{F_{l+1}^{s}}/{(2l+3)}=0\Rightarrow \nu _{l}^{\pm l}=0$, i.e., the |*m*| = *l* components will be completely suppressed in low energies, suggesting a hierarchy structure for gapless QSLs in three dimensions.
- Both zero and first sounds can be studied via the minimal models that are of either two channel or three channel.
  - The results have been summarized in Table 1
  - For first sound, the only weakly damped mode is the longitudinal mode.
  - When the electronic liquid becomes incompressible, the first sound velocity will behave quite differently under two incompressibility conditions: (i) for $1+{F_{1}^{s}}/{d}=0$, *c*_1_ → 0, while for (ii) $F_{0}^{s}\rightarrow +\infty$, *c*_1_ → +∞.

Indeed, it was pointed out in [15] that the usual *U*(1) QSL state can be realized by the Landau-type effective theory by keeping scatterings in *l* = 0, 1 channels only. The gauge (or transverse current) fluctuations in the *U*(1) QSL are nothing but *l* = 1 and *m* = ±1 zero sound modes studied in the present work. What is more, our theory suggests that a gapless QSL may possess a spinon Fermi surface that breaks the *SO*(3) rotational symmetry. Such a generic spinon Fermi surface can be characterized by the hierarchy structure of the Landau parameters and zero sound modes [see (27)].

We emphasize that first sounds are ordinary sound modes that even exist in a free Fermi gas. These first sound modes can be measured by usual ultrasonic techniques, because despite being charge neutral, the elementary excitations, spinons, couple to phonons in exactly the same way that electrons do in the long-wavelength limit [23]. But zero sound modes do not have their non-interacting correspondence, and exist only in specific regions of the interacting parameters. Experimentally, the velocity and attenuation of zero sounds can be measured via the acoustic impedance and other methods in analogy to liquid ^3^He [24,25].

In addition to incompressible electronic liquids studied in this work, collective effects have been widely studied in diverse aspects of physics too, including Weyl semi-metal [26], cold atoms [27,28], soft matters [29], astrophysics [30], high-energy physics [31] and high-pressure physics [32]. We expect that our research will generate further research interests in related fields.

## METHOD

### Functions for type-I algebraic equations

For zero sound in three dimensions, $\Omega _{ll^{\prime }}^{m}$, $\Theta _{l}^{m}$ and $\alpha _{l}^{m}$ are defined as


(50a) \begin{eqnarray*} \Omega _{ll^{\prime }}^{m}(s) &=& \Omega _{l^{\prime }l}^{m}(s) \\ &=& \frac{1}{2}\sqrt{\frac{(l-m)!\, (l^{\prime }-m)!}{(l+m)!\, (l^{\prime }+m)!}} \\ &&\quad \times \int _{-1}^{1}d\mu P_{l}^{m}(\mu ) \frac{\mu }{\mu -s}P_{l^{\prime }}^{m}(\mu ),\\ \end{eqnarray*}

(50b) \begin{eqnarray*} \Theta _{l}^{m}(s) &=& (-1)^{m}\Theta _{l}^{-m}(s) \\ &=& \frac{1}{2}\sqrt{\frac{(l-m)!}{(l+m)!}}\int _{-1}^{1}d\mu P_{l}^{m}(\mu )\frac{\mu }{\mu -s}\\ \end{eqnarray*}

and


(50c) \begin{eqnarray*} \alpha _{l}^{m}&=& (-1)^{m}\alpha _{l}^{-m} \\ &=& \frac{2l+1}{2}\sqrt{\frac{(l-m)!}{(l+m)!}}\int _{-1}^{1}d\mu P_{l}^{m}(\mu )\mu ,\\ \end{eqnarray*}

respectively, where the $P_{l}^{m}$ are associated Legendre polynomials, and $\mu = {\rm cos\theta p}$. To derive equations (13), we have used the following relations: (1) $\Omega _{ll^{\prime }}^{m}\left(s\right)=0$ for *m* > *l, l*′; (2) $\Theta _{l}^{m}\left(s\right)=0$ for *m* > *l*, (3) $\Theta ^0_l(s)=\Omega ^0_{l0}(s)$ and (4) $\alpha _{l}^{m}=0$ when *l* − *m* is even and $\alpha _{l}^{0}=0$ for *l* > 1.

In two dimensions, $\Pi _{ll^{\prime }}(s)=\Pi _{l^{\prime }l}(s)$ and $\Xi _{ll^{\prime }}(s)=\Xi _{l^{\prime }l}(s)$ are defined as


(51) \begin{eqnarray*} \Pi _{ll^{\prime }}(s)&&=-\int \frac{d\theta }{2\pi }\cos (l\theta ) \frac{\cos \theta }{s-\cos \theta }\cos (l^{\prime }\theta ), \\ \Xi _{ll^{\prime }}(s)&&=-\int \frac{d\theta }{2\pi }\sin (l\theta ) \frac{\cos \theta }{s-\cos \theta }\sin (l^{\prime }\theta ). \\ \end{eqnarray*}

### Secular equations of minimal models

In the minimal models, the algebraic equations form a closed set of linear equations. The mode frequency is determined by the secular equation, that is, the determinant of the coefficients of linear equations equals zero. By analyzing the limits of the determinant, we can obtain the sufficient condition for a real solution *s* > 1 to the secular equation

#### Longitudinal mode in three dimensions

The algebraic equations in the minimal model are given by


(52) \begin{eqnarray*} && \left(\begin{array}{ccc} 1+F_{0}^{s}\Omega _{00} & F_{1}^{s}\Omega _{01} & F_{2}^{s}\Omega _{02}\\ F_{0}^{s}\Omega _{10} & 1+F_{1}^{s}\Omega _{11} & F_{2}^{s}\Omega _{12}\\ F_{0}^{s}\Omega _{20} & F_{1}^{s}\Omega _{21} & 1+F_{2}^{s}\Omega _{22} \end{array}\right) \left(\begin{array}{c}\tilde{\nu }_{0}\\ \tilde{\nu }_{1}\\ \tilde{\nu }_{2} \end{array}\right) \\ &&\qquad = -U \left(\begin{array}{c}\Omega _{00}\\ \Omega _{10}\\ \Omega _{20} \end{array}\right), \end{eqnarray*}

where $\tilde{\nu }_{l}\equiv {\nu _{l}}/{(2l+1)} $. Then the mode frequency is determined by the secular equation


(53) \begin{eqnarray*} &&\det \left(\begin{array}{ccc} 1+F_{0}^{s}\Omega _{00} & F_{1}^{s}\Omega _{01} & F_{2}^{s}\Omega _{02} \\ F_{0}^{s}\Omega _{10} & 1+F_{1}^{s}\Omega _{11} & F_{2}^{s}\Omega _{12} \\ F_{0}^{s}\Omega _{20} & F_{1}^{s}\Omega _{21} & 1+F_{2}^{s}\Omega _{22} \end{array}\right)\\ &&\qquad \equiv g_{3}(s)=0, \end{eqnarray*}

which is written explicitly in (17). Note that *g*_3_(*s* → ∞) = 1 > 0 and


\begin{eqnarray*} g_{3}(s\rightarrow {1})&=& \bigg \lbrace \bigg ( 1+\frac{F_{1}^{s}}{3}\bigg )\\ && \times \, \bigg [ \frac{3+F_{0}^{s}}{2}+\frac{F_2^{s}}{20}(1-3F_{0}^{s})\bigg ] \\ &&+\, \frac{9}{20}\bigg (F_{2}^{s}-\frac{10}{3}\bigg )\bigg \rbrace \\&&\ln \frac{s-1}{2} + O(1). \end{eqnarray*}

Therefore, the inequality


\begin{eqnarray*} &&\bigg (1+\frac{F_{1}^{s}}{3}\bigg )\bigg [\frac{3+F_{0}^{s}}{2}+\frac{F_{2}^{s}}{20} (1-3F_{0}^{s})\bigg ]\\ &&\qquad +\, \frac{9}{20}\bigg (F_{2}^{s}-\frac{10}{3}\bigg ) >0, \end{eqnarray*}

which is equivalent to (18), gives rise to a sufficient condition for a real solution *s* > 1 to the secular equation *g*_3_(*s*) = 0.

#### The *m* ≥ 1 zero sound modes

The algebraic equations are given by


(54) \begin{eqnarray*} && \left({\begin{array}{cc} 1+F_{m}^{s}\Omega _{mm}^{m} & F_{m+1}^{s}\Omega _{mm+1}^{m}\\ F_{m}^{s}\Omega _{m+1m}^{m} & 1+F_{m+1}^{s}\Omega _{m+1m+1}^{m} \end{array}} \right) \left({\begin{array}{c}\tilde{\nu }_{m}^{m}\\ \tilde{\nu }_{m+1}^{m} \end{array}}\right) \\ &&\qquad = -U \left({\begin{array}{c}\Theta _{m}^{m}\\ \Theta _{m+1}^{m} \end{array}}\right), \end{eqnarray*}

where $\tilde{\nu }_{m}^{m}={\nu _{m}^{m}}/{(2m+1)}$. The corresponding secular equation is


(55) \begin{eqnarray*} &&\det \left({\begin{array}{cc}1+F_{m}^{s}\Omega _{mm}^{m} & F_{m+1}^{s}\Omega _{mm+1}^{m}\\ F_{m}^{s}\Omega _{m+1m}^{m} & 1+F_{m+1}^{s}\Omega _{m+1m+1}^{m} \end{array}}\right)\\ &&\qquad\quad \equiv h(s)=0, \end{eqnarray*}

which is written explicitly in (20) using the relations in Table 2. Note that *h*(*s* → ∞) = 1 and


(56) \begin{eqnarray*} h(s\rightarrow {}1) &=& -\frac{1}{2m(2m+3)} \\ &&\times \, \bigg [2+\bigg (1+\frac{F_{m}^{s}}{2m+1}\bigg )\bigg ]F_{m+1}^{s}\\ &&+\, \frac{1}{2m}\bigg [2m+1-\bigg (1+\frac{F_{m}^{s}}{2m+1}\bigg )\bigg ].\\ \end{eqnarray*}

Since $1+{F_{m}^{s}}/{(2m+1)}\ge 0$ is required by thermodynamic stability of the system [17,18], there must exist a real solution *s*(>1) for *h*(*s*) = 0, as long as


(57) \begin{eqnarray*} F_{m+1}^{s}>(2m+3)\frac{2m+1-\left[1+{F_{m}^{s}}/{(2m+1)}\right]}{2+\left[1+{F_{m}^{s}}/{(2m+1)}\right]}.\!\!\!\!\!\!\!\!\\ \end{eqnarray*}

#### Longitudinal mode in two dimensions

The algebraic equations and secular equation are given by


(58) \begin{eqnarray*} && \left({\begin{array}{ccc}1+F_{0}^{s}\Pi _{00} & F_{1}^{s}\Pi _{01} & F_{2}^{s}\Pi _{02}\\ F_{0}^{s}\Pi _{10} & 1+F_{1}^{s}\Pi _{11} & F_{2}^{s}\Pi _{12}\\ F_{0}^{s}\Pi _{20} & F_{1}^{s}\Pi _{21} & 1+F_{2}^{s}\Pi _{22} \end{array}}\right) {\left(\begin{array}{c}u_{0}\\ {u_{1}}/{2}\\ {u_{2}}/{2} \end{array}\right)} \\ &&\qquad = -U \left({\begin{array}{c}\Pi _{00}\\ \Pi _{10}\\ \Pi _{20} \end{array}}\right) \end{eqnarray*}

and


(59) \begin{eqnarray*} &&\det \left({\begin{array}{ccc}1+F_{0}^{s}\Pi _{00} & F_{1}^{s}\Pi _{01} & F_{2}^{s}\Pi _{02}\\ F_{0}^{s}\Pi _{10} & 1+F_{1}^{s}\Pi _{11} & F_{2}^{s}\Pi _{12}\\ F_{0}^{s}\Pi _{20} & F_{1}^{s}\Pi _{21} & 1+F_{2}^{s}\Pi _{22} \end{array}}\right)\\ &&\qquad\quad \equiv h_{3}(s)=0, \end{eqnarray*}

which is written explicitly in (42). Note that *h*_3_(*s* → ∞) = 1 > 0 and


\begin{eqnarray*} h_{3}(s\rightarrow 1) &=& -\bigg \lbrace \bigg (1+\frac{F_{1}^{s}}{2}\bigg ) \bigg [2+F_{0}^{s}-\frac{F_{2}^{s}}{2}F_{0}^{s}\bigg ] \\ && +\, 2\bigg (\frac{F_{2}^{s}}{2}-1 \! \bigg )\!\bigg \rbrace \frac{1}{\sqrt{2(s-1)}}+O(1). \end{eqnarray*}

Therefore, the inequality


\begin{eqnarray*} &&\bigg (1+\frac{F_{1}^{s}}{2}\bigg )\bigg [2+F_{0}^{s}-\frac{F_{2}^{s}}{2}F_{0}^{s}\bigg ] \\ && \quad +\, 2\bigg (\frac{F_{2}^{s}}{2}-1\bigg ) >0, \end{eqnarray*}

which is equivalent to (43), gives rise to a sufficient condition for a real solution *s* > 1 to the secular equation *h*_3_(*s*) = 0.

#### Transverse mode in two dimensions

The algebraic equations and secular equation are given by


(60) \begin{eqnarray*} &&\left( {\begin{array}{cc}1+F_{1}^{s}\Xi _{11} & F_{2}^{s}\Xi _{12}\\ F_{1}^{s}\Xi _{21} & 1+F_{2}^{s}\Xi _{22} \end{array}} {\begin{array}{c}v_{1}/{2}\\ {v_{2}}/{2} \end{array}}\right) \\ &&\qquad = -U \left({\begin{array}{c}\Xi _{11}\\ \Xi _{21} \end{array}}\right) \end{eqnarray*}

and


(61) \begin{eqnarray*} \det \left({\begin{array}{cc}1+F_{1}^{s}\Xi _{11} & F_{2}^{s}\Xi _{12}\\ F_{1}^{s}\Xi _{21} & 1+F_{2}^{s}\Xi _{22} \end{array}}\right) \equiv \bar{h}_{2}(s)=0,\\ \end{eqnarray*}

which is written explicitly in (45) using the relations in Table 2. Note that $\bar{h}_{2}(s\rightarrow \infty )=2F_{2}^{s}s^{2}$ and


(62) \begin{eqnarray*} \bar{h}_{2}(s\rightarrow 1)=\frac{1}{2}\frac{F_{1}^{s}(1+{F_{2}^{s}}/{2}) +4F_{2}^{s}}{\sqrt{2(s-1)}}+O(1).\\ \end{eqnarray*}

The sufficient condition of a real solution *s*(>1) for $\bar{h}_{2}(s)=0$ is


(63) \begin{eqnarray*} F_{2}^{s}\bigg [F_{1}^{s}\bigg (1+\frac{F_{2}^{s}}{2}\bigg )+4F_{2}^{s}\bigg ]<0. \end{eqnarray*}

## Supplementary Material

nwac251_Supplemental_File
