# Supplementary material for: Collective effects in an incompressible electronic liquid
Source: Natl Sci Rev. 2022 Oct 23;10(6):nwac251. doi: 10.1093/nsr/nwac251 (PMC10171675; doi:10.1093/nsr/nwac251)
Supplement: nwac251_Supplemental_File [file nwac251_supplemental_file.pdf]

Supplementary Materials for "Collective effects in an incompressible electronic liquid"

Jian-Jian Miao,[1] Hui-Ke Jin,[2] and Yi Zhou[3,4,5,*]

[1]*Department of Physics, The Chinese University of Hong Kong, Shatin, New Territories, Hong Kong, China*
[2]*Department of Physics TQM, Technische Universität München, James-Franck-Straße 1, D-85748 Garching, Germany*
[3]*Institute of Physics, Chinese Academy of Sciences, Beijing 100190, China*
[4]*Songshan Lake Materials Laboratory, Dongguan, Guangdong 523808, China*
[5]*Kavli Institute for Theoretical Sciences & CAS Center for Excellence in Topological Quantum Computation, University of Chinese Academy of Sciences, Beijing 100190, China*
(Dated: October 25, 2022)

This is to provide more details for the main text.

* yizhou@iphy.ac.cn

Appendix A: Zero sound: longitudinal ($m = 0$) mode in 3D

In this appendix, we shall derive the algebraic equations for the longitudinal model in the collisionless regime. With the help of the addition theorem,

$$P_l\left(\cos\theta_{\mathrm{pp}'}\right)=\sum_{m=-l}^{l}Y_l^m\left(\theta_{\mathrm{p}},\phi_{\mathrm{p}}\right)Y_l^{m*}\left(\theta_{\mathrm{p}'},\phi_{\mathrm{p}'}\right),$$

we have

$$\begin{aligned}
&\sum_{\mathrm{p}'}f^s_{\mathrm{pp}'}\frac{\partial n^0_{\mathrm{p}'}}{\partial\varepsilon_{\mathrm{p}'}}\nu_{\mathrm{p}'}\\
=&N(0)\int d\varepsilon\int\frac{d\hat{\mathrm{p}}'}{4\pi}f^s_{\mathrm{pp}'}\frac{\partial n^0_\varepsilon}{\partial\varepsilon}\nu_{\mathrm{p}'}\\
=&-N(0)\int\frac{d\hat{\mathrm{p}}'}{4\pi}\sum_{l,l'}P_l\left(\cos\theta_{\mathrm{pp}'}\right)f^s_lP_{l'}\left(\cos\theta_{\mathrm{p}'}\right)\nu_{l'}\\
=&-\int\frac{d\hat{\mathrm{p}}'}{4\pi}\sum_{l,l'}\sum_{m=-l}^{l}Y_l^m\left(\theta_{\mathrm{p}},\phi_{\mathrm{p}}\right)Y_l^{m*}\left(\theta_{\mathrm{p}'},\phi_{\mathrm{p}'}\right)F^s_lP_{l'}\left(\cos\theta_{\mathrm{p}'}\right)\nu_{l'}\\
=&-\sum_{l,l'}\sum_{m=-l}^{l}Y_l^m\left(\theta_{\mathrm{p}},\phi_{\mathrm{p}}\right)F^s_l\int\frac{d\hat{\mathrm{p}}'}{4\pi}Y_l^{m*}\left(\theta_{\mathrm{p}'},\phi_{\mathrm{p}'}\right)Y^0_{l'}\left(\theta_{\mathrm{p}'},\phi_{\mathrm{p}'}\right)\nu_{l'}\\
=&-\sum_{l=0}^{\infty}\sum_{m=-l}^{l}Y_l^m\left(\theta_{\mathrm{p}},\phi_{\mathrm{p}}\right)F^s_l\sum_{l'=0}^{\infty}\frac{1}{2l+1}\delta_{ll'}\delta_{m0}\nu_{l'}\\
=&-\sum_{l=0}^{\infty}Y^0_l\left(\theta_{\mathrm{p}},\phi_{\mathrm{p}}\right)F^s_l\frac{\nu_l}{2l+1}\\
=&-\sum_{l=0}^{\infty}F^s_lP_l\left(\cos\theta_{\mathrm{p}}\right)\frac{\nu_l}{2l+1}.
\end{aligned}$$

Putting the above into Eq. (??) leads to

$$\sum_l\nu_lP_l(\mu)-\frac{\mu}{s-\mu}\sum_lF^s_l\frac{1}{2l+1}\nu_lP_l(\mu)=\frac{\mu}{s-\mu}U,\quad\text{(A1)}$$

where $s\equiv\frac{\omega}{qv_F}$ and $\mu=\cos\theta_{\mathrm{p}}$. Using the orthogonal relations,

$$\int_{-1}^{1}d\mu P_l(\mu)P_{l'}(\mu)=\frac{2}{2l+1}\delta_{ll'},$$

we can rewrite Eq. (A1) into Eq. (??).

On the other hand, by using Bonnet's recursion formula

$$\mu P_l(\mu)=\frac{1}{2l+1}\left[(l+1)P_{l+1}(\mu)+lP_{l-1}(\mu)\right],$$

we also have

$$\begin{aligned}
\mu U=&\sum_l\nu_l\left[s-\left(1+\frac{F^s_l}{2l+1}\right)\mu\right]P_l(\mu)\\
=&\sum_lP_l(\mu)\left[\nu_ls-\left(1+\frac{F^s_{l-1}}{2l-1}\right)\frac{l}{2l-1}-\left(1+\frac{F^s_{l+1}}{2l+3}\right)\frac{l+1}{2l+3}\right].
\end{aligned}$$

This leads another set of algebraic equations, i.e., Eq. (??), as

$$\nu_ls-\left(1+\frac{F^s_{l-1}}{2l-1}\right)\frac{l}{2l-1}\nu_{l-1}-\left(1+\frac{F^s_{l+1}}{2l+3}\right)\frac{l+1}{2l+3}\nu_{l+1}=\delta_{l1}U.\quad\text{(A2)}$$

For later convenience, here we write down some useful relations for $\Omega_{ll'}(s)$, i.e. $\Omega_{ll'}(s)$ can be expressed directly in terms of the first and second kind of Legendre functions, P_l and Q_l, as

$$\Omega_{l'l}=\Omega_{ll'}=\frac{\delta_{ll'}}{2l+1}-sP_{l'}(s)Q_l(s),\ l'\leq l.\quad\text{(A3)}$$

1. Algebraic equations of type I and type II

In the main text, we obtain the type I algebraic equation [i.e., Eq. (??)] as

$$\frac{\nu_l}{2l+1}+\sum_{l'=0}^{\infty}F^s_{l'}\Omega_{ll'}\frac{\nu_{l'}}{2l'+1}=-\Omega_{l0}U,\quad\text{(A4)}$$

and the type II algebraic equation [i.e., Eq. (??)] as

$$\nu_ls-l\left(1+\frac{F^s_{l-1}}{2l-1}\right)\frac{\nu_{l-1}}{2l-1}-(l+1)\left(1+\frac{F^s_{l+1}}{2l+3}\right)\frac{\nu_{l+1}}{2l+3}=\delta_{l1}U,\quad\text{(A5)}$$

which are both derived from the Landau kinetic equation in the collisionless regime and thus are equivalent. It's an easy exercise that one can derive algebraic equation type I from algebraic equation type II by multiplying the Legendre polynomials, summing over all components, and using Bonnet's recursion formula for Legendre polynomials.

The algebraic equations type I and type II are two representations of Landau kinetic equation in the collisionless regime. The former is in the infinitely coupled form and can be solved by truncation in Landau parameters F^s_l, while the later is in the hierarchical from. In order to manifest the equivalence, multiplying the $l=0$ and $l=1$ components of algebraic equation I by Ω_{10} and Ω_{00}, respectively, and meanwhile using the relations $\Omega_{l1}=s\Omega_{l0}+\frac{1}{3}\delta_{l1}$, we can eliminate the U terms and obtain

$$\begin{aligned}
0=&\left(\nu_0+\sum_{l'=0}^{\infty}F^s_{l'}\Omega_{0l'}\frac{\nu_{l'}}{2l'+1}\right)\Omega_{10}-\left(\frac{\nu_1}{3}+\sum_{l'=0}^{\infty}F^s_{l'}\Omega_{1l'}\frac{\nu_{l'}}{2l'+1}\right)\Omega_{00}\\
=&\Omega_{10}\nu_0-\Omega_{00}\left(1+\frac{F^s_1}{3}\right)\frac{\nu_1}{3}=\Omega_{00}\left[\nu_0s-\left(1+\frac{F^s_1}{3}\right)\frac{\nu_1}{3}\right],
\end{aligned}$$

which is nothing but the $l=0$ component of type II algebraic equation,

$$\nu_0s-\left(1+\frac{F^s_1}{3}\right)\frac{\nu_1}{3}=0.\quad\text{(A6)}$$

2. Two channel model

We consider a two channel model by keeping only F^s_0 and F^s_1 and setting $F^s_l=0$ for $l\geq 2$. Introducing a new variable

$\tilde{\nu}_l \equiv \frac{\nu_l}{2l+1}$ for convenience, one can simplify Eq. (**??**) as follows [Eq. (**??**)],

$$\tilde{\nu}_l + F_0^s \Omega_{l0}(s)\tilde{\nu}_0 + F_1^s \Omega_{l1}(s)\tilde{\nu}_1 = -\Omega_{l0}(s)U. \tag{A7}$$

The above consists of a series of algebraic equations that can be classified into two categories: (i) $l \geq 2$ and (ii) $l = 0$ and $l = 1$. The equations in the second category form a close set which is composed of $\tilde{\nu}_0$ and $\tilde{\nu}_1$:

$$\begin{pmatrix} 1 + F_0^s \Omega_{00} & F_1^s \Omega_{01} \\ F_0^s \Omega_{10} & 1 + F_1^s \Omega_{11} \end{pmatrix} \begin{pmatrix} \tilde{\nu}_0 \\ \tilde{\nu}_1 \end{pmatrix} = -U \begin{pmatrix} \Omega_{00} \\ \Omega_{10} \end{pmatrix}. \tag{A8}$$

The solution to above equations leads to the response functions as follows,

$$\begin{aligned} \frac{\tilde{\nu}_0}{U} &= -\frac{\left(1 + F_1^s \Omega_{11}\right)\Omega_{00} - F_1^s \Omega_{01}\Omega_{10}}{\left(1 + F_0^s \Omega_{00}\right)\left(1 + F_1^s \Omega_{11}\right) - F_0^s \Omega_{10} F_1^s \Omega_{01}} \\ &= -\frac{\left(1 + \frac{F_1^s}{3}\right)\Omega_{00}}{\left(1 + F_0^s \Omega_{00}\right)\left(1 + \frac{F_1^s}{3}\right) + F_1^s s^2 \Omega_{00}}, \end{aligned} \tag{A9a}$$

$$\begin{aligned} \frac{\tilde{\nu}_1}{U} &= -\frac{-F_0^s \Omega_{10}\Omega_{00} + \left(1 + F_0^s \Omega_{00}\right)\Omega_{10}}{\left(1 + F_0^s \Omega_{00}\right)\left(1 + F_1^s \Omega_{11}\right) - F_0^s \Omega_{10} F_1^s \Omega_{01}} \\ &= -\frac{\Omega_{10}}{\left(1 + F_0^s \Omega_{00}\right)\left(1 + \frac{F_1^s}{3}\right) + F_1^s s^2 \Omega_{00}}. \end{aligned} \tag{A9b}$$

From the above, we find that a complete suppression of $\tilde{\nu}_0$ component requires that

$$\left(1 + \frac{F_1^s}{3}\right)\Omega_{00} = 0. \tag{A10}$$

As the solution to $\Omega_{00}(s) = 0$ is $s = \infty$, $1 + \frac{1}{3}F_1^s = 0$ is nothing but the QSL condition given in Eq. (**??**).

We proceed to consider the possibility of the complete suppression of $\tilde{\nu}_1$ component, which leads to

$$\Omega_{10} = s\Omega_{00} = 0. \tag{A11}$$

The solutions are $s = 0$ and $s = \infty$. This means that it is impossible to suppress the $l = 1$ component entirely, and such a mode is generally allowed.

The mode frequency can be determined via the pole of $\tilde{\nu}_l$ from Eqs. (A9), i.e.,

$$1 + \frac{F_1^s}{3} + \Omega_{00}\left[F_0^s\left(1 + \frac{F_1^s}{3}\right) + F_1^s s^2\right] = 0. \tag{A12}$$

To find a real solution s to the above equation, we define a function

$$g_2(s) = 1 + \frac{F_1^s}{3} + \Omega_{00}\left[F_0^s\left(1 + \frac{F_1^s}{3}\right) + F_1^s s^2\right],$$

and have $g_2(s \to \infty) = 1 > 0$. Meanwhile, when $s \to 1$, the leading term in $g_2(s)$ reads

$$g_2(s \to 1) = \frac{1}{2}\left[F_0^s\left(1 + \frac{F_1^s}{3}\right) + F_1^s\right]\ln\frac{s-1}{2} + O(1).$$

Therefore, to have a real solution for $g_2(s) = 0$, one requires that

$$F_0^s\left(1 + \frac{F_1^s}{3}\right) + F_1^s > 0. \tag{A13}$$

Eq. (A12) must generally be found numerically. Using asymptotic expressions for $\Omega_{00}(s)$ as

$$\Omega_{00}(s) = \begin{cases} 1 + \frac{1}{2}\ln\frac{s-1}{2}, & s \to 1 + 0^+, \\ -\frac{1}{3s^2} - \frac{1}{5s^4} - \frac{1}{7s^6} - \cdots, & s \to \infty, \end{cases}$$

one can find that Eq. (A12) can be approximately solved in two limits

$$s = \begin{cases} 1 + 2e^{-2\left[1 + \frac{1 + \frac{F_1^s}{3}}{F_0^s\left(1 + \frac{F_1^s}{3}\right) + F_1^s}\right]}, & 0 < s - 1 \ll 1, \\ \sqrt{\frac{F_0^s}{3}\left(1 + \frac{F_1^s}{3}\right) + \frac{F_1^s}{5}}, & s \to \infty. \end{cases} \tag{A14}$$

Note that in the weak coupling limit $F_1^s \ll F_0^s \ll 1$, the zero sound velocity is $c_0 = sv_F \approx v_F$, and meanwhile the first sound velocity is $c_1 = v_F\sqrt{\frac{1}{3}\left(1 + F_0^s\right)\left(1 + \frac{F_1^s}{3}\right)} \approx v_F/\sqrt{3} \approx c_0/\sqrt{3}$.

Under the condition of QSL in 2D, Eq. (A12) reduces to

$$s^2\Pi_{00}(s) = 0. \tag{A15}$$

There is no nontrivial real solution, indicating the absence of weakly damped zero sound mode in the two channel QSL model. Also, when $1 + \frac{F_1^s}{3} \to 0^+$, the above inequality Eq. (A13) can not be satisfied unless $F_0^s \to +\infty$. In order to obtain a weakly damped longitudinal zero sound mode in a finite F_0^s and under the QSL condition $1 + \frac{F_1^s}{3} = 0^+$, one need to involve more Landau parameters F_l^s with $l \geq 2$.

3. Three channel model

Here we provide some details for solving the secular equation $g_3(s) = 0$, i.e., Eq. (**??**) in the main text. To do this, we expand $g_3(s)$ around $s = 1$ and $s = \infty$. With the help of the following relations:

$$\begin{aligned} \Omega_{02}(s) &= \frac{1}{2} + P_2(s)\Omega_{00}(s), \\ \Omega_{22}(s) &= \frac{1}{5} + P_2(s)\Omega_{02}, \\ P_2(s) &= \frac{1}{2}(3s^2 - 1), \end{aligned}$$

we can rewrite Eq. (**??**) as

$$\begin{aligned} g_3(s) = &\left(1 + \frac{F_1^s}{3}\right)\left(1 + F_2^s \Omega_{22}\right) \\ &+ \left[F_0^s\left(1 + \frac{F_1^s}{3}\right) + F_1^s s^2\right]\left[\Omega_{00} + F_2^s\left(\Omega_{00}\Omega_{22} - \Omega_{02}^2\right)\right] \\ = &A(s) + B(s)\Omega_{00}(s) = \tilde{A}(s) + \tilde{B}(s)\ln\frac{s-1}{s+1}. \end{aligned} \tag{A16}$$

Here

$$A(s) = A_2 s^2 + A_0 = \frac{3}{4}F_2^s s^2 + \left(1+\frac{F_1^s}{3}\right)\left[1-\frac{F_2^s}{20}\left(1+5F_0^s\right)\right], \quad \text{(A17)}$$

$$B(s) = B_4 s^4 + B_2 s^2 + B_0 = \frac{9}{4}F_2^s s^4 - 3s^2\left\{\frac{F_2^s}{20}\left[9+\left(1+5F_0^s\right)\left(1+\frac{F_1^s}{3}\right)\right]-\frac{F_1^s}{3}\right\} + \left[F_2^s\left(\frac{1}{4}+\frac{9F_0^s}{20}\right)+F_0^s\right]\left(1+\frac{F_1^s}{3}\right), \quad \text{(A18)}$$

and

$$\tilde{A}(s) = A(s) + B(s), \quad \text{(A19)}$$

$$\tilde{B}(s) = \frac{s}{2}B(s), \quad \text{(A20)}$$

where A_0, A_2, B_0, B_2 and B_4 are coefficients that does not depend on s. Thus the secular equation becomes

$$A(s) + B(s)\Omega_{00}(s) = \tilde{A}(s) + \tilde{B}(s)\ln\frac{s-1}{s+1} = 0. \quad \text{(A21)}$$

First, we consider the solution $s \to 1+0^+$. When

$$\frac{\tilde{A}(s\to 1+0^+)}{\tilde{B}(s\to 1+0^+)} = \frac{11}{3} + \mathbb{C} \gg 1 \quad \text{(A22)}$$

with $\mathbb{C} = \frac{50-\left(30+\frac{50F_0^s+8F_2^s}{3}\right)\left(1+\frac{F_1^s}{3}\right)}{F_2^s\left[9+\left(1-3F_0^s\right)\left(1+\frac{F_1^s}{3}\right)\right]+10\left[\left(3+F_0^s\right)\left(1+\frac{F_1^s}{3}\right)-3\right]}$, the secular equation has an approximate solution

$$s \simeq 1 + 2\exp\left[-\frac{\tilde{A}(s=1)}{\tilde{B}(s=1)}\right] = 1 + 2\exp\left\{-\frac{11}{3}-\mathbb{C}\right\}. \quad \text{(A23)}$$

Second, we look for the solution $s \to +\infty$, which is available when

$$A_2 = \frac{B_4}{3},$$

and

$$\frac{\frac{B_4}{7}+\frac{B_2}{5}+\frac{B_0}{3}}{A_0-\frac{B_2}{3}-\frac{B_4}{5}} = \frac{B_4}{7}+\frac{B_2}{5}+\frac{B_0}{3} \gg 1.$$

In this situation, we have another solution

$$s \simeq \sqrt{\frac{B_4}{7}+\frac{B_2}{5}+\frac{B_0}{3}} = \sqrt{\frac{F_2^s}{25}\left[\frac{9}{7}+\frac{4}{3}\left(1+\frac{F_1^s}{3}\right)\right]+\frac{F_0^s}{3}\left(1+\frac{F_1^s}{3}\right)+\frac{F_1^s}{5}}. \quad \text{(A24)}$$

Appendix B: Zero sound: generic m modes in 3D

In consistence with the $m=0$ case, we choose the following definition for spherical harmonics,

$$Y_l^m(\theta,\phi) = \sqrt{\frac{(l-m)!}{(l+m)!}}P_l^m(\cos\theta)e^{im\phi},$$

$$\int\frac{d\hat{n}}{4\pi}Y_l^m(\theta,\phi)Y_{l'}^{m'*}(\theta,\phi) = \frac{\delta_{ll'}\delta_{mm'}}{2l+1},$$

where P_l^m are associated Legendre functions satisfying the relation

$$P_l^{-m}(x) = (-1)^m\frac{(l-m)!}{(l+m)!}P_l^m(x).$$

Similarly, we have

$$\sum_{p'} f_{pp'}^s\frac{\partial n_{p'}^0}{\partial\varepsilon_{p'}}\nu_{p'} = -\sum_l F_l^s\frac{1}{2l+1}\sum_{m=-l}^{l}Y_l^m(\theta_p,\phi_p)\nu_l^m,$$

and

$$\sum_l \nu_l^m Y_l^m(\theta_p,\phi_p) - \frac{\cos\theta_p}{s-\cos\theta_p}\sum_l F_l^s\frac{1}{2l+1}\nu_l^m Y_l^m(\theta_p,\phi_p) = \frac{\cos\theta_p}{s-\cos\theta_p}Ue^{im\phi_p}. \quad \text{(B1)}$$

Then, by defining

$$\begin{aligned}\Omega_{ll'}^m(s) &= \Omega_{l'l}^m(s)\\ &= -\int\frac{d\hat{p}}{4\pi}Y_l^{m*}\left(\theta_p,\phi_p\right)\frac{\cos\theta_p}{s-\cos\theta_p}Y_{l'}^m\left(\theta_p,\phi_p\right)\\ &= \frac{1}{2}\sqrt{\frac{(l-m)!}{(l+m)!}\frac{(l'-m)!}{(l'+m)!}}\int_{-1}^{1}d\mu P_l^m(\mu)\frac{\mu}{\mu-s}P_{l'}^m(\mu),\end{aligned} \quad \text{(B2)}$$

as well as

$$\begin{aligned}\Theta_l^m(s) &= (-1)^m\Theta_l^{-m}(s)\\ &= -\int\frac{d\hat{p}}{4\pi}Y_l^{m*}\left(\theta_p,\phi_p\right)\frac{\cos\theta_p}{s-\cos\theta_p}e^{im\phi_p}\\ &= -\int\frac{d\hat{p}}{4\pi}\sqrt{\frac{(l-m)!}{(l+m)!}}P_l^m\left(\cos\theta_p\right)e^{-im\phi_p}\frac{\cos\theta_p}{s-\cos\theta_p}e^{im\phi_p}\\ &= \frac{1}{2}\sqrt{\frac{(l-m)!}{(l+m)!}}\int_{-1}^{1}d\mu P_l^m(\mu)\frac{\mu}{\mu-s},\end{aligned} \quad \text{(B3)}$$

we obtain type I algebraic equations similar to Eq. (**??**) as follows,

$$\frac{\nu_l^m}{2l+1} + \sum_{l'=m}^{\infty}F_{l'}^s\Omega_{ll'}^m(s)\frac{\nu_{l'}^m}{2l'+1} = -\Theta_l^m(s)\,U. \quad \text{(B4)}$$

Here we have used the facts that $\Omega_{ll'}^m(s) = 0$ for $m > l, l'$ and $\Theta_l^m(s) = 0$ for $m > l$.

We can further write Eq. (B1) in terms of associated Legendre functions as

$$\sum_l \nu_l^m \sqrt{\frac{(l-m)!}{(l+m)!}} P_l^m(\mu) - \frac{\mu}{s-\mu}\sum_l F_l^s \frac{1}{2l+1}\nu_l^m \sqrt{\frac{(l-m)!}{(l+m)!}} P_l^m(\mu) = \frac{\mu}{s-\mu}U. \tag{B5}$$

Using the following formula,

$$\begin{aligned}(2l+1)\mu P_l^m(\mu) &= (l-m+1)P_{l+1}^m(\mu) + (l+m)P_{l-1}^m(\mu),\\ \int_{-1}^1 d\mu P_l^m(\mu)P_{l'}^m(\mu) &= \frac{2}{2l+1}\frac{(l+m)!}{(l-m)!}\delta_{ll'},\end{aligned}$$

we can obtain

$$\begin{aligned}\mu U &= \sum_l \nu_l^m \left[s - \left(1+\frac{F_l^s}{2l+1}\right)\mu\right]\sqrt{\frac{(l-m)!}{(l+m)!}}P_l^m(\mu)\\
&= \sum_l \nu_l^m s\sqrt{\frac{(l-m)!}{(l+m)!}}P_l^m(\mu) - \sum_l \nu_l^m\left(1+\frac{F_l^s}{2l+1}\right)\times\\
&\quad \sqrt{\frac{(l-m)!}{(l+m)!}}\frac{1}{2l+1}\left[(l-m+1)P_{l+1}^m(\mu) + (l+m)P_{l-1}^m(\mu)\right]\\
&= \sum_l \nu_l^m s\sqrt{\frac{(l-m)!}{(l+m)!}}P_l^m(\mu) - \sum_l \nu_l^m\left(1+\frac{F_l^s}{2l+1}\right)\times\\
&\quad \frac{\sqrt{(l+1)^2-m^2}}{2l+1}\sqrt{\frac{(l-m+1)!}{(l+m+1)!}}P_{l+1}^m(\mu) -\\
&\quad \sum_l \nu_l^m\left(1+\frac{F_l^s}{2l+1}\right)\frac{\sqrt{l^2-m^2}}{2l+1}\sqrt{\frac{(l-m-1)!}{(l+m-1)!}}P_{l-1}^m(\mu)\\
&= \sum_l \sqrt{\frac{(l-m)!}{(l+m)!}}P_l^m(\mu)\tilde{F}_l^m\Big[\nu_l^m s - \nu_{l-1}^m\left(1+\frac{F_{l-1}^s}{2l-1}\right)\frac{\sqrt{l^2-m^2}}{2l-1}\\
&\quad - \nu_{l+1}^m\left(1+\frac{F_{l+1}^s}{2l+3}\right)\frac{\sqrt{(l+1)^2-m^2}}{2l+3}\Big].\end{aligned}$$

This leads to the type II algebraic equations which are similar to Eq. (??)

$$\nu_l^m s - \nu_{l-1}^m\left(1+\frac{F_{l-1}^s}{2l-1}\right)\frac{\sqrt{l^2-m^2}}{2l-1} - \nu_{l+1}^m\left(1+\frac{F_{l+1}^s}{2l+3}\right)\frac{\sqrt{(l+1)^2-m^2}}{2l+3} = \alpha_l^m U, \tag{B6}$$

where

$$\alpha_l^m = (-1)^m\alpha_l^{-m} = \frac{2l+1}{2}\sqrt{\frac{(l-m)!}{(l+m)!}}\int_{-1}^1 d\mu P_l^m(\mu)\mu.$$

Note that $P_l^m(\mu) = (-1)^m(1-\mu^2)^{m/2}\frac{d^m}{d\mu^m}P_l(\mu)$ are even (odd) functions of μ when $l-m$ are even (odd). So that $\alpha_l^m = 0$ when $m > l$ or $l-m$ is even. Moreover, we also have $\alpha_l^0 = 0$ for $l > 1$, and $\alpha_l^{-m} = (-1)^m\alpha_l^m$.

1. Some useful formula

In the collisionless regime, we obtain the type I and type II algebraic equations from Landau kinetic for $m > 0$, in which we introduce following three functions

$$\Omega_{ll'}^m(s) = \frac{1}{2}\sqrt{\frac{(l-m)!}{(l+m)!}\frac{(l'-m)!}{(l'+m)!}}\int_{-1}^1 d\mu P_l^m(\mu)\frac{\mu}{\mu-s}P_{l'}^m(\mu), \tag{B7}$$

$$\Theta_l^m(s) = \frac{1}{2}\sqrt{\frac{(l-m)!}{(l+m)!}}\int_{-1}^1 d\mu P_l^m(\mu)\frac{\mu}{\mu-s}, \tag{B8}$$

$$\alpha_l^m = \frac{2l+1}{2}\sqrt{\frac{(l-m)!}{(l+m)!}}\int_{-1}^1 d\mu P_l^m(\mu)\mu, \tag{B9}$$

where the associated Legendre functions are given by

$$P_l^m(\mu) = \frac{(-1)^m}{2^l l!}\left(1-\mu^2\right)^{m/2}\frac{d^{l+m}}{d\mu^{l+m}}\left(\mu^2-1\right)^l. \tag{B10}$$

Since $P_l^m(\mu) = 0$ for $m > l$, we have $\Omega_{ll'}^m(s) = 0$ for $m > l, l'$, $\Theta_l^m(s) = \alpha_l^m = 0$ for $m > l$. From the relations between $P_l^{-m}(\mu)$ and $P_l^m(\mu)$,

$$P_l^{-m}(\mu) = (-1)^m\frac{(l-m)!}{(l+m)!}P_l^m(\mu), \tag{B11}$$

we can obtain

$$\Omega_{ll'}^m(s) = \Omega_{ll'}^{-m}(s), \tag{B12}$$
$$\Theta_l^m(s) = (-1)^m\Theta_l^{-m}(s), \tag{B13}$$
$$\alpha_l^m = (-1)^m\alpha_l^{-m}. \tag{B14}$$

By definition, $\Omega_{ll'}^m$ is symmetric in terms of two subscripts l and l' as

$$\Omega_{ll'}^m(s) = \Omega_{l'l}^m(s). \tag{B15}$$

Using the identities of associated Legendre functions as

$$P_m^m(\mu) = P_m^m(-\mu) = (-1)^m(2m-1)!!\left(1-\mu^2\right)^{m/2}, \tag{B16}$$
$$P_{m+1}^m(\mu) = \mu(2m+1)P_m^m(\mu), \tag{B17}$$
$$P_{m+1}^{m+1}(\mu) = -(2m+1)\sqrt{1-\mu^2}P_m^m(\mu), \tag{B18}$$

the orthogonality of associated Legendre functions for fixed m as

$$\int_{-1}^1 d\mu P_k^m(\mu)P_l^m(\mu) = \frac{2(l+m)!}{(2l+1)(l-m)!}\delta_{kl}, \tag{B19}$$

and the integrals of associated Legendre functions and polynomials as

$$\frac{1}{2}\frac{1}{(2m)!}\int_{-1}^1 d\mu\left[P_m^m(\mu)\right]^2\mu^{2n} = \frac{(2m-1)!!(2n-1)!!}{(2m+2n+1)!!}, \tag{B20}$$

we can obtain useful relations of $\Omega^m_{ll'}$ function

$$\Omega^m_{m+1,l}(s) = s\sqrt{2m+1}\Omega^m_{ml}(s) + \frac{\delta_{m+1,l}}{(2m+3)}, \tag{B21}$$

$$\Omega^{m+1}_{m+1,m+1}(s) = \frac{2m+1}{2m+2}\left(1-s^2\right)\Omega^m_{mm}(s) - \frac{1}{(2m+2)(2m+3)}. \tag{B22}$$

For later use, the large s limit of $\Omega^m_{mm}(s)$ is given by

$$\begin{aligned}\lim_{s\to\infty}\Omega^m_{mm}(s) &= \lim_{s\to\infty}\frac{1}{2}\frac{1}{(2m)!}\int_{-1}^{1}d\mu\left[P^m_m(\mu)\right]^2\frac{\mu}{\mu-s}\\ &= -\frac{1}{2}\frac{1}{(2m)!}\int_{-1}^{1}d\mu\left[P^m_m(\mu)\right]^2\sum_{n=1}^{\infty}\left(\frac{\mu}{s}\right)^{2n}\\ &= -\sum_{n=1}^{\infty}\frac{(2m-1)!!\,(2n-1)!!}{(2m+2n+1)!!}\frac{1}{s^{2n}}.\end{aligned} \tag{B23}$$

For $\Theta^m_l(s)$, we similarly obtain the useful relations as follows

$$\Theta^m_{m+1}(s) = s\sqrt{2m+1}\Theta^m_m(s), \tag{B24}$$

$$\Theta^{2m}_{2m}(s) = \sqrt{\frac{(4m-1)!!}{(4m)!!}}\frac{(2m)!!}{(2m-1)!!}\Omega^m_{mm}(s). \tag{B25}$$

For the α^m_l function, from the parity of associated Legendre functions

$$P^m_l(-\mu) = (-1)^{l+m}P^m_l(\mu), \tag{B26}$$

we have $\alpha^m_l = 0$ for $l+m =$ even. From the relation between Legendre polynomials and associated Legendre polynomials as

$$P^0_l(\mu) = P_l(\mu), \tag{B27}$$

and the orthogonality of Legendre polynomials as

$$\int_{-1}^{1}d\mu P_l(\mu)P_{l'}(\mu) = \frac{2}{2l+1}\delta_{ll'}, \tag{B28}$$

we can obtain that $\alpha^0_l = 0$ for $l > 1$.

We can use these relations to prove that the $l = m$ and $l = m+1$ components of type I algebraic equation can lead to the $l = m$ component of type II algebraic equation as follows

$$\begin{aligned}0 =& -\Theta^m_m U\Theta^m_{m+1} + \Theta^m_{m+1}U\Theta^m_m\\ =&\left(\frac{\nu^m_m}{2m+1} + \sum_{l'=m}^{\infty}F^s_{l'}\Omega^m_{ml'}\frac{\nu^m_{l'}}{2l'+1}\right)\Theta^m_{m+1} - \left(\frac{\nu^m_{m+1}}{2m+3} + \sum_{l'=m}^{\infty}F^s_{l'}\Omega^m_{m+1,l'}\frac{\nu^m_{l'}}{2l'+1}\right)\Theta^m_m\\ =&\left[(1+F^s_m\Omega^m_{mm})\Theta^m_{m+1} - F^s_m\Omega^m_{m+1m}\Theta^m_m\right]\frac{\nu^m_m}{2m+1} + \left[F^s_{m+1}\Omega^m_{mm+1}\Theta^m_{m+1} - \left(1+F^s_{m+1}\Omega^m_{m+1m+1}\right)\Theta^m_m\right]\frac{\nu^m_{m+1}}{2m+3}\\ &+\sum_{l'=m+2}^{\infty}F^s_{l'}\left(\Omega^m_{ml'}\Theta^m_{m+1} - \Omega^m_{m+1,l'}\Theta^m_m\right)\frac{\nu^m_{l'}}{2l'+1}\\ =&\left[(1+F^s_m\Omega^m_{mm})s\sqrt{2m+1} - F^s_m\Omega^m_{m+1m}\right]\Theta^m_m\frac{\nu^m_m}{2m+1} + \left[F^s_{m+1}\Omega^m_{mm+1}s\sqrt{2m+1} - \left(1+F^s_{m+1}\Omega^m_{m+1m+1}\right)\right]\Theta^m_m\frac{\nu^m_{m+1}}{2m+3}\\ =&\left\{\left[s\sqrt{2m+1} + F^s_m\Omega^m_{mm}\left(s\sqrt{2m+1} - s\sqrt{2m+1}\right)\right]\frac{\nu^m_m}{2m+1} + \left[-1 + F^s_{m+1}\left(\Omega^m_{mm+1}s\sqrt{2m+1} - \Omega^m_{m+1m+1}\right)\right]\frac{\nu^m_{m+1}}{2m+3}\right\}\Theta^m_m\\ =&\left\{\frac{\nu^m_m s}{\sqrt{2m+1}} + \left[-1 - \frac{F^s_{m+1}}{2m+3}\right]\frac{\nu^m_{m+1}}{2m+3}\right\}\Theta^m_m\\ \Longrightarrow&\nu^m_m s - \sqrt{2m+1}\left(1 + \frac{F^s_{m+1}}{2m+3}\right)\frac{\nu^m_{m+1}}{2m+3} = 0.\end{aligned} \tag{B29}$$

Appendix C: Zero sound: two channel model for $m > 0$ modes in 3D

The type I algebraic equations for generic m modes are

$$\frac{\nu^m_l}{2l+1} + \sum_{l'=0}^{\infty}F^s_{l'}\Omega^m_{ll'}(s)\frac{\nu^m_{l'}}{2l'+1} = -\Theta^m_l(s)U,$$

while the type II algebraic equation read

$$\nu^m_l s - \sqrt{l^2-m^2}\left(1+\frac{F^s_{l-1}}{2l-1}\right)\frac{\nu^m_{l-1}}{2l-1} - \sqrt{(l+1)^2-m^2}\left(1+\frac{F^s_{l+1}}{2l+3}\right)\frac{\nu^m_{l+1}}{2l+3} = \alpha^m_l U.$$

It turns out that there exists a specific solution for a given good quantum number m and the two channel model.

To find this specific solution, we consider a two channel model by keeping the Landau parameters up to F^s_{m+1}, i.e., $F^s_l = 0$ for $l > m+2$. Then the $l = m$ component of the type I equation reduces to

$$\frac{\nu^m_m}{2m+1} + F^s_m \Omega^m_{mm} \frac{\nu^m_m}{2m+1} + F^s_{m+1} \Omega^m_{mm+1} \frac{\nu^m_{m+1}}{2m+3} = -\Theta^m_m U. \tag{C1}$$

The $l = m$ component of type II equation is given by

$$\nu^m_m s - \sqrt{2m+1}\left(1 + \frac{F^s_{m+1}}{2m+3}\right)\frac{\nu^m_{m+1}}{2m+3} = 0. \tag{C2}$$

Those two equations (similar to the two channel model for $m = 0$ modes) form a close set and can be easily solved as follows,

$$\frac{\nu^m_m}{U} = -\frac{\left(1 + \frac{F^s_{m+1}}{2m+3}\right)\Theta^m_m}{\frac{(1+F^s_m\Omega^m_{mm})\left(1+\frac{F^s_{m+1}}{2m+3}\right)}{2m+1} + \frac{F^s_{m+1}\Omega^m_{mm+1}s}{\sqrt{2m+1}}}, \tag{C3}$$

$$\frac{\nu^m_{m+1}}{2m+3} = \frac{\nu^m_m s}{\sqrt{2m+1}\left(1 + \frac{F^s_{m+1}}{2m+3}\right)} = -\frac{s\Theta^m_m U}{\frac{(1+F^s_m\Omega^m_{mm})\left(1+\frac{F^s_{m+1}}{2m+3}\right)}{\sqrt{2m+1}} + F^s_{m+1}\Omega^m_{mm+1}s}. \tag{C4}$$

The mode frequency is determined by the pole of the response function

$$(1 + F^s_m\Omega^m_{mm})\left(1 + \frac{F^s_{m+1}}{2m+3}\right) + \sqrt{2m+1}F^s_{m+1}\Omega^m_{mm+1}s = 0. \tag{C5}$$

Using the relation derived from Eq. (B21)

$$\Omega^m_{mm+1}(s) = \sqrt{2m+1}\, s\,\Omega^m_{mm}(s), \tag{C6}$$

the equation determining mode frequency is simplified as

$$[1 + F^s_m\Omega^m_{mm}(s)]\left(1 + \frac{F^s_{m+1}}{2m+3}\right) + (2m+1)F^s_{m+1}s^2\Omega^m_{mm}(s) = 0. \tag{C7}$$

1. $m = 1$ modes

For the $m = 1$ modes, we obtain

$$\frac{\nu^1_1}{U} = -\frac{3\left(1 + \frac{F^s_2}{5}\right)\Theta^1_1}{\left(1 + F^s_1\Omega^1_{11}\right)\left(1 + \frac{F^s_2}{5}\right) + 3F^s_2\Omega^1_{11}s^2}. \tag{C8}$$

The ν^1_1 mode is completely suppressed when

$$\left(1 + \frac{F^s_2}{5}\right)\Theta^1_1(s) = 0. \tag{C9}$$

As there is no real solution to $\Theta^1_1(s) = 0$ except at $s \to \infty$, thus we obtain $1 + \frac{F^s_2}{5} = 0$, which is nothing but the Pomeranchuk instability in $l = 2$ channel.

The frequency of $m = 1$ mode is determined by

$$\left(1 + F^s_1\Omega^1_{11}\right)\left(1 + \frac{F^s_2}{5}\right) + 3F^s_2\Omega^1_{11}s^2 = 0. \tag{C10}$$

The above equation must generally be found numerically. Using the limiting form $\lim_{s\to\infty}\Omega^1_{11}(s) = -\frac{1}{15s^2} - \frac{1}{35s^4}$, Eq. (C10) can approximately be solved in the large s limit

$$s = \sqrt{\frac{F^s_1}{15}\left(1 + \frac{F^s_2}{5}\right) + \frac{3F^s_2}{35}}. \tag{C11}$$

Note that the above solution is well defined only when $\frac{F^s_1}{15}\left(1 + \frac{F^s_2}{5}\right) + \frac{3F^s_2}{35} \gg 1$.

From Eq. (**??**) and the inequality $1 + \frac{F^s_1}{3} \geq 0$, we have the following condition for a real solution $s > 1$ to the frequency equation,

$$F^s_2 > \frac{5}{2 + \left(1 + \frac{F^s_1}{3}\right)}\left[3 - \left(1 + \frac{F^s_1}{3}\right)\right]. \tag{C12}$$

In the limit of $1 + \frac{F^s_1}{3} = 0$, the above inequality becomes

$$F^s_2 > \frac{15}{2}. \tag{C13}$$

For a solution $s \to 1 + 0^+$, it is hard to obtain a simple analytical form, since the convergence of $\lim_{s\to 1}(s-1)\ln(s-1) = 0$ is very slow. The numerical solution to $h(s) = 0$ can be found in Fig. **??** (b) as a 3D contour plot in the parameter space $\left(s, 1 + \frac{F^s_1}{3}, \frac{2F^s_2}{15}\right)$.

2. $m = 2$ modes

For the $m = 2$ modes, we obtain

$$\frac{\nu^2_2}{U} = -\frac{5\left(1 + \frac{F^s_3}{7}\right)\Theta^2_2}{\left(1 + F^s_2\Omega^2_{22}\right)\left(1 + \frac{F^s_3}{7}\right) + 5F^s_3\Omega^2_{22}s^2}. \tag{C14}$$

The ν^2_2 mode is completely suppressed when

$$\left(1 + \frac{F^s_3}{7}\right)\Theta^2_2(s) = 0. \tag{C15}$$

As there is no real solution to $\Theta^2_2(s) = 0$ except at $s \to \infty$, thus we obtain $1 + \frac{F^s_3}{7} = 0$, which is nothing but the Pomeranchuk instability for $l = 3$ channel.

The mode frequency of ν^2_2 mode is determined by

$$\left(1 + F^s_2\Omega^2_{22}\right)\left(1 + \frac{F^s_3}{7}\right) + 5F^s_3\Omega^2_{22}s^2 = 0. \tag{C16}$$

The above equation must generally be found numerically. Using the limiting form $\lim_{s\to\infty}\Omega_{22}^{2}(s)=-\frac{1}{35s^2}-\frac{1}{105s^4}$, Eq. (C10) can approximately be solved in the large s limit

$$s=\sqrt{\frac{F_2^s}{35}\left(1+\frac{F_3^s}{7}\right)+\frac{F_3^s}{21}}, \tag{C17}$$

when the condition

$$\frac{F_2^s}{35}\left(1+\frac{F_3^s}{7}\right)+\frac{F_3^s}{21s^2}\gg 1 \tag{C18}$$

is satisfied.

From Eq. (??) and the inequality $1+\frac{F_2^s}{5}\geq 0$, we obtain the following condition for a real solution $s>1$ to the frequency equation,

$$F_2^s>\frac{7}{2+\left(1+\frac{F_2^s}{5}\right)}\left[5-\left(1+\frac{F_1^s}{5}\right)\right]. \tag{C19}$$

In the limit of $1+\frac{F_2^s}{5}=0$, the above inequality becomes

$$F_3^s>\frac{35}{2}. \tag{C20}$$

For a solution $s\to 1+0^+$, it is hard to obtain a simple analytical form, since the convergence of $\lim_{s\to 1}(s-1)\ln(s-1)=0$ is very slow. The numerical solution to $h(s)=0$ can be found in Fig. ?? (b) as a 3D contour plot in the parameter space $\left(s,1+\frac{F_2^s}{5},\frac{2F_3^s}{35}\right)$.

3. Arbitrary $m\geq 1$ modes

The mode frequency of ν_m^m mode is determined by

$$\left(1+F_m^s\Omega_{mm}^m\right)\left(1+\frac{F_{m+1}^s}{2m+3}\right)+(2m+1)\,F_{m+1}^s\Omega_{mm}^m s^2=0. \tag{C21}$$

Using the limiting form $\lim_{s\to\infty}\Omega_{mm}^m(s)=-\frac{1}{(2m+1)(2m+3)s^2}-\frac{3}{(2m+1)(2m+3)(2m+5)}\frac{1}{s^4}$, the solution to the above equation in the large s limit is

$$s=\sqrt{\frac{F_m^s}{(2m+1)(2m+3)}\left(1+\frac{F_{m+1}^s}{2m+3}\right)+\frac{3F_{m+1}^s}{(2m+3)(2m+5)}}. \tag{C22}$$

when the condition

$$\frac{F_m^s}{(2m+1)(2m+3)}\left(1+\frac{F_{m+1}^s}{2m+3}\right)+\frac{3F_{m+1}^s}{(2m+3)(2m+5)}\gg 1 \tag{C23}$$

is satisfied.

Appendix D: Zero sound in 2D: Algebraic equations for longitudinal and transverse modes

To derive algebraic equations for collective modes in 2D, we consider the expansion for the quasi-particle interaction $f_{pp'}^s$ in Eq. (??b). With the expressions for ν_p in Eq. (??) and (??), we have

$$\begin{aligned}&\sum_{p'}f_{pp'}^s\frac{\partial n_{p'}^0}{\partial\varepsilon_{p'}}\nu_{p'}\\ &=N(0)\int d\varepsilon\frac{\partial n^0(\varepsilon)}{\partial\varepsilon}\int\frac{d\theta_{p'}}{2\pi}f_{pp'}^s\nu_{p'}\\ &=-N(0)\int\frac{d\theta_{p'}}{2\pi}\sum_{l=0}^{\infty}f_l^s\cos(l\theta_{pp'})\sum_{l'=-\infty}^{\infty}\nu_{l'}e^{il'\theta_{p'}}\\ &=-\frac{1}{2}\int\frac{d\theta_{p'}}{2\pi}\sum_{l=0}^{\infty}F_l^s\left[e^{il(\theta_p-\theta_{p'})}+e^{-il(\theta_p-\theta_{p'})}\right]\sum_{l'=-\infty}^{\infty}\nu_{l'}e^{il'\theta_{p'}}\\ &=-\frac{1}{2}\sum_{l=0}^{\infty}F_l^s\left(\nu_l e^{il\theta_p}+\nu_{-l}e^{-il\theta_p}\right).\end{aligned}$$

Putting the above into Eq. (??) leads to

$$\begin{aligned}&\sum_{m=-\infty}^{\infty}\nu_m e^{im\theta_p}-\frac{1}{2}\frac{\cos\theta_p}{s-\cos\theta_p}\sum_{l=0}^{\infty}F_l^s\left(\nu_l e^{il\theta_p}+\nu_{-l}e^{-il\theta_p}\right)\\ &=\frac{\cos\theta_p}{s-\cos\theta_p}U.\end{aligned} \tag{D1}$$

For the longitudinal mode ν_p^+, Eq. (D1) becomes

$$\begin{aligned}&\sum_{l=0}^{\infty}u_l\cos(l\theta_p)-\frac{\cos\theta_p}{s-\cos\theta_p}\left[F_0^s u_0+\frac{1}{2}\sum_{l=1}^{\infty}F_l^s u_l\cos(l\theta_p)\right]\\ &=\frac{\cos\theta_p}{s-\cos\theta_p}U,\end{aligned} \tag{D2}$$

while for the transverse mode ν_p^-, by using Eqs. (??) and (??), we have

$$\begin{aligned}&\sum_{l=1}^{\infty}\nu_l\sin(l\theta_p)-\frac{1}{2}\frac{\cos\theta_p}{s-\cos\theta_p}\sum_{l=1}^{\infty}F_l^s\nu_l\sin(l\theta_p)\\ &=\frac{\cos\theta_p}{s-\cos\theta_p}U\sin\theta_p.\end{aligned} \tag{D3}$$

Using the orthogonal relations for cosine functions with $l,l'\geq 0$,

$$\int\frac{d\theta_p}{2\pi}\cos\left(l\theta_p\right)\cos\left(l'\theta_p\right)=\frac{1}{2}\left(\delta_{ll'}+\delta_{l0}\delta_{l'0}\right) \tag{D4}$$

the type I algebraic equation for longitudinal mode is derived from Eq. (D2) as follows

$$\frac{u_l+\delta_{l0}u_0}{2}+\sum_{l'=0}^{\infty}F_{l'}^s\Pi_{ll'}(s)\frac{u_{l'}+\delta_{l'0}u_0}{2}=-\Pi_{l0}(s)\,U, \tag{D5}$$

where

$$\Pi_{ll'}(s)=-\int\frac{d\theta_p}{2\pi}\cos\left(l\theta_p\right)\frac{\cos\theta_p}{s-\cos\theta_p}\cos\left(l'\theta_p\right) \tag{D6}$$

with $\Pi_{ll'}(s) = \Pi_{l'l}(s)$ Using orthogonal relations for sine functions with $l, l' \geq 0$

$$\int \frac{d\theta_p}{2\pi} \sin\left(l\theta_p\right) \sin\left(l'\theta_p\right) = \frac{1}{2}\left(\delta_{ll'} - \delta_{l0}\delta_{l'0}\right), \tag{D7}$$

the type I algebraic equation for transverse mode is derived from Eq. (D3) as follows

$$\frac{v_l}{2} + \sum_{l'=0}^{\infty} F^s_{l'} \Xi_{ll'}(s) \frac{v_{l'}}{2} = -\Xi_{l1}(s) U, \tag{D8}$$

where

$$\Xi_{ll'}(s) = -\int \frac{d\theta_p}{2\pi} \sin\left(l\theta_p\right) \frac{\cos\theta_p}{s - \cos\theta_p} \sin\left(l'\theta_p\right) \tag{D9}$$

with $\Xi_{ll'}(s) = \Xi_{l'l}(s)$.

From Eq. (D2), we obtain that

$$\begin{aligned}
\cos\theta_p U &= \left(s - \cos\theta_p\right) \sum_{l=0}^{\infty} \cos\left(l\theta_p\right) u_l - \cos\theta_p \sum_{l=0}^{\infty} F^s_l \cos\left(l\theta_p\right) \frac{u_l + \delta_{l0}u_0}{2} \\
&= \sum_{l=0}^{\infty} \cos\left(l\theta_p\right) u_l s - \cos\theta_p \left(1 + F^s_0\right) u_0 - \sum_{l=1}^{\infty} \left(1 + \frac{F^s_l}{2}\right) \cos\theta_p \cos\left(l\theta_p\right) u_l \\
&= \sum_{l=0}^{\infty} \cos\left(l\theta_p\right) u_l s - \cos\theta_p \left(1 + F^s_0\right) u_0 - \sum_{l=1}^{\infty} \left(1 + \frac{F^s_l}{2}\right) \cos\left(l\theta_p + \theta_p\right) \frac{u_l}{2} - \sum_{l=1}^{\infty} \left(1 + \frac{F^s_l}{2}\right) \cos\left(l\theta_p - \theta_p\right) \frac{u_l}{2} \\
&= \tilde{u}_0 s - \left(1 + \frac{F^s_1}{2}\right)\frac{u_1}{2} + \sum_{l=1}^{\infty} \cos\left(l\theta_p\right) \left[u_l s - \left(1 + \frac{F^s_{l-1} + \delta_{l1}F^s_0}{2}\right) \frac{u_{l-1} + \delta_{l1}u_0}{2} - \left(1 + \frac{F^s_{l+1}}{2}\right)\frac{u_{l+1}}{2}\right].
\end{aligned} \tag{D10}$$

Thus the type II algebraic equation for the longitudinal mode is

$$u_l s - \left(1 + \frac{F^s_{l-1} + \delta_{l1}F^s_0}{2}\right) \frac{u_{l-1} + \delta_{l1}u_0}{2} - \left(1 + \frac{F^s_{l+1}}{2}\right)\frac{u_{l+1}}{2} = \delta_{l1} U.$$

In particular, for $l = 0$, we have

$$u_0 s - \left(1 + \frac{F^s_1}{2}\right)\frac{u_1}{2} = 0. \tag{D11}$$

In general dimensions, we conjecture that the $l = 0$ component of type II algebraic equation for longitudinal mode is

$$v_0 s - \left(1 + \frac{F^s_1}{d}\right)\frac{v_1}{d} = 0. \tag{D12}$$

From Eq. (D3), we obtain that

$$\begin{aligned}
&\cos\theta_p \sin\theta_p U = \sin 2\theta_p \frac{U}{2} \\
&= \left(s - \cos\theta_p\right) \sum_{l=1}^{\infty} v_l \sin\left(l\theta_p\right) - \cos\theta_p \left[\sum_{l=1}^{\infty} F^s_l \frac{v_l}{2} \sin\left(l\theta_p\right)\right] \\
&= \sum_{l=1}^{\infty} \sin\left(l\theta_p\right) \left[v_l s - \left(1 + \frac{F^s_{l-1}}{2}\right)\frac{v_{l-1}}{2} - \left(1 + \frac{F^s_{l+1}}{2}\right)\frac{v_{l+1}}{2}\right].
\end{aligned}$$

By noting that $v_0 = 0$, the type II algebraic equation for transverse mode is found to be

$$v_l s - \left(1 + \frac{F^s_{l-1}}{2}\right)\frac{v_{l-1}}{2} - \left(1 + \frac{F^s_{l+1}}{2}\right)\frac{v_{l+1}}{2} = \delta_{l2}\frac{U}{2}. \tag{D13}$$

Appendix E: Zero sound: longitudinal mode in 2D

The algebraic equations type I and II for longitudinal mode in the collisionless regime in 2D are given by

$$\frac{u_l + \delta_{l0}u_0}{2} + \sum_{l'=0}^{\infty} F^s_{l'} \Pi_{ll'}(s) \frac{u_{l'} + \delta_{l'0}u_0}{2} = -\Pi_{l0}(s) U,$$

and

$$u_l s - \left(1 + \frac{F^s_{l-1} + \delta_{l1}F^s_0}{2}\right) \frac{u_{l-1} + \delta_{l1}u_0}{2} - \left(1 + \frac{F^s_{l+1}}{2}\right)\frac{u_{l+1}}{2} = \delta_{l1} U,$$

respectively. These two algebraic equations are equivalent. In order to manifest the equivalence, by using the following relations $\Pi_{l1} - s\Pi_{l0} = \frac{1}{2}\delta_{l1}$, and then multiplying the $l = 0$ and $l = 1$ components of algebraic equation type I by Π_{10} and Π_{00}, respectively, we can eliminate the U terms and obtain

$$\begin{aligned}
0 &= \left(u_0 + \sum_{l'=0}^{\infty} F^s_{l'} \Pi_{0l'}(s) \frac{u_{l'} + \delta_{l'0}u_0}{2}\right) \Pi_{10} \\
&\quad - \left(\frac{u_1}{2} + \sum_{l'=0}^{\infty} F^s_{l'} \Pi_{1l'}(s) \frac{u_{l'} + \delta_{l'0}u_0}{2}\right) \Pi_{00} \\
&= \Pi_{10} u_0 - \Pi_{00}\left(1 + \frac{F^s_1}{2}\right)\frac{u_1}{2} = \Pi_{00}\left[u_0 s - \left(1 + \frac{F^s_1}{2}\right)\frac{u_1}{2}\right],
\end{aligned}$$

which is nothing but the $l = 0$ component of algebraic equation type II.

1. Two channel model

Now we consider a two channel model by keeping only F_0^s and F_1^s and setting $F_l^s = 0$ for $l \geq 2$. The components of algebraic equation I can be classified into two categories: (i) $l \geq 2$ and (ii) $l = 0$ and $l = 1$. The components in the second category form a close set that is composed of u_0 and u_1:

$$u_0 + F_0^s \Pi_{00} u_0 + F_1^s \Pi_{01} \frac{u_1}{2} = -\Pi_{00} U, \tag{E1}$$

$$\frac{u_1}{2} + F_0^s \Pi_{10} u_0 + F_1^s \Pi_{11} \frac{u_1}{2} = -\Pi_{10} U. \tag{E2}$$

The solution from algebraic equation I leads to the response functions as follows,

$$\begin{aligned} \frac{u_0}{U} &= -\frac{\left(1 + F_1^s \Pi_{11}\right) \Pi_{00} - F_1^s \Pi_{01} \Pi_{10}}{\left(1 + F_0^s \Pi_{00}\right)\left(1 + F_1^s \Pi_{11}\right) - F_0^s \Pi_{10} F_1^s \Pi_{01}} \\ &= -\frac{\left(1 + \frac{F_1^s}{2}\right) \Pi_{00}}{\left(1 + F_0^s \Pi_{00}\right)\left(1 + \frac{F_1^s}{2}\right) + F_1^s s^2 \Pi_{00}}, \\ \frac{u_1}{2U} &= -\frac{-F_0^s \Pi_{10} \Pi_{00} + \left(1 + F_0^s \Pi_{00}\right) \Pi_{10}}{\left(1 + F_0^s \Pi_{00}\right)\left(1 + F_1^s \Pi_{11}\right) - F_0^s \Pi_{10} F_1^s \Pi_{01}} \\ &= -\frac{s \Pi_{00}}{\left(1 + F_0^s \Pi_{00}\right)\left(1 + \frac{F_1^s}{2}\right) + F_1^s s^2 \Pi_{00}}, \end{aligned} \tag{E3}$$

where we have used the relations $\Pi_{l1} = s\Pi_{l0} + \frac{1}{2}\delta_{l1}$.

From the above solution, we find that the complete suppression of u_0 component requires that

$$\left(1 + \frac{F_1^s}{2}\right) \Pi_{00}(s) = 0. \tag{E4}$$

As there is no real solution to $\Pi_{00}(s) = 0$ except at $s \to \infty$, thus we obtain $1 + \frac{F_1^s}{2} = 0$, which is nothing but the QSL condition in 2D as given in Eq. (**??**).

We proceed to consider the possibility of the complete suppression of u_1 component, which leads to

$$s \Pi_{00}(s) = 0. \tag{E5}$$

Again there is no real solution except at $s = 0$ or $s \to \infty$. This means that it is impossible to suppress the $l = 1$ component entirely, and such a mode is generally allowed.

The zero sound mode frequency can be determined via the pole of the response functions, i.e.,

$$\left(1 + F_0^s \Pi_{00}\right)\left(1 + \frac{F_1^s}{2}\right) + F_1^s s^2 \Pi_{00} = 0. \tag{E6}$$

To find a real solution s to the above equation, we define function

$$h_2(s) = \left(1 + \frac{F_1^s}{2}\right) + \Pi_{00}\left[F_0^s\left(1 + \frac{F_1^s}{2}\right) + F_1^s s^2\right]. \tag{E7}$$

Note that $h_2(s \to \infty) = 1 > 0$ and meanwhile

$$h_2(s \to 1) = -\frac{F_0^s\left(1 + \frac{F_1^s}{2}\right) + F_1^s}{\sqrt{2(s-1)}} + O(1). \tag{E8}$$

To have a real solution for $h_2(s) = 0$, one requires

$$F_0^s\left(1 + \frac{F_1^s}{2}\right) + F_1^s > 0. \tag{E9}$$

Eq. (E6) must generally be found numerically. Using the limiting forms of Π_{00},

$$\Pi_{00}(s) = \begin{cases} 1 - \frac{1}{\sqrt{2(s-1)}}, & 0 < s-1 \ll 1, \\ -\frac{1}{2s^2} - \frac{3}{8s^4} - \frac{5}{16s^6} - \cdots, & s \to \infty, \end{cases} \tag{E10}$$

Eq. (E6) can approximately be solved in two limits

$$s = \begin{cases} 1 + \frac{1}{2\left[1 + \frac{\left(1 + \frac{F_1^s}{2}\right)}{F_0^s\left(1 + \frac{F_1^s}{2}\right) + F_1^s}\right]^2}, & 0 < s-1 \ll 1, \\ \sqrt{\frac{F_0^s}{2}\left(1 + \frac{F_1^s}{2}\right) + \frac{3F_1^s}{8}}, & s \to \infty. \end{cases} \tag{E11}$$

Under the condition of QSL in 2D, Eq. (E6) reduces to

$$s^2 \Pi_{00}(s) = 0. \tag{E12}$$

There is no nontrivial real solution, indicating the absence of weakly damped zero sound mode in the two channel QSL model. Also, when $1 + \frac{F_1^s}{2} \to 0^+$, the inequality Eq. (E9) can not be satisfied unless $F_0^s \to \infty$. In order to obtain a weakly damped longitudinal zero sound mode in a finite F_0^s and under the QSL condition $1 + \frac{F_1^s}{2} \to 0^+$, one need to involve more F_l^s with $l \geq 2$.

2. Three channel model

Consider a three channel model by keeping F_0^s, F_1^s and F_2^s and setting $F_l^s = 0$ for $l \geq 3$, we obtain the close set of components v_0, v_1, and v_2 from algebraic equation I as follows,

$$\begin{pmatrix} 1 + F_0^s \Pi_{00} & F_1^s \Pi_{01} & F_2^s \Pi_{02} \\ F_0^s \Pi_{10} & 1 + F_1^s \Pi_{11} & F_2^s \Pi_{12} \\ F_0^s \Pi_{20} & F_1^s \Pi_{21} & 1 + F_2^s \Pi_{22} \end{pmatrix} \begin{pmatrix} u_0 \\ \frac{u_1}{2} \\ \frac{u_2}{2} \end{pmatrix} = -U \begin{pmatrix} \Pi_{00} \\ \Pi_{10} \\ \Pi_{20} \end{pmatrix}. \tag{E13}$$

Then the mode frequency is determined by the following secular equation,

$$h_3(s) = \det \begin{pmatrix} 1 + F_0^s \Pi_{00} & F_1^s \Pi_{01} & F_2^s \Pi_{02} \\ F_0^s \Pi_{10} & 1 + F_1^s \Pi_{11} & F_2^s \Pi_{12} \\ F_0^s \Pi_{20} & F_1^s \Pi_{21} & 1 + F_2^s \Pi_{22} \end{pmatrix} = 0. \tag{E14}$$

Here the function $h_3(s)$ can be written explicitly as follows,

$$h_3(s) = \left(1+\frac{F_1^s}{2}\right)\left(1+F_2^s\Pi_{22}\right)+\left[F_0^s\left(1+\frac{F_1^s}{2}\right)+F_1^s s^2\right]\left[\Pi_{00}+F_2^s\left(\Pi_{00}\Pi_{22}-\Pi_{20}^2\right)\right]. \tag{E15}$$

To find a real solution s to the secular equation, we notice that $h_3(s\to\infty)=1>0$ and

$$h_3(s\to 1) = -\left\{\left(1+\frac{F_1^s}{2}\right)\left[2+F_0^s-\frac{F_2^s}{2}F_0^s\right]+2\left(\frac{F_2^s}{2}-1\right)\right\}\frac{1}{\sqrt{2(s-1)}}. \tag{E16}$$

Therefore, the inequality

$$\left(1+\frac{F_1^s}{2}\right)\left[2+F_0^s-\frac{F_2^s}{2}F_0^s\right]+2\left(\frac{F_2^s}{2}-1\right)>0, \tag{E17}$$

or

$$\left[1-\frac{F_0^s}{2}\left(1+\frac{F_1^s}{2}\right)\right]F_2^s+\left[F_0^s\left(1+\frac{F_1^s}{2}\right)+F_1^s\right]>0, \tag{E18}$$

gives rise to a sufficient condition for a real solution $s>1$ to the secular equation $h_3(s)=0$.

In general, the secular equation $h_3(s)=0$ must be found numerically. Nevertheless we can solve the secular equation analytically around $s=1$ and $s=\infty$. To do this, we expand $h_3(s)$ around $s=1$ and $s=\infty$. With the help of the following relations:

$$\Pi_{20} = 1+\left(2s^2-1\right)\Pi_{00}, \tag{E19}$$

$$\Pi_{22} = 2s^2-\frac{1}{2}+\left(2s^2-1\right)^2\Pi_{00}, \tag{E20}$$

we can rewrite $h_3(s)$ as follows,

$$\begin{aligned} h_3(s) &= \left(1+\frac{F_1^s}{2}\right)\left(1+F_2^s\Pi_{22}\right)+\left[F_0^s\left(1+\frac{F_1^s}{2}\right)+F_1^s s^2\right]\left[\Pi_{00}+F_2^s\left(\Pi_{00}\Pi_{22}-\Pi_{20}^2\right)\right] \\ &= C(s)+D(s)\Pi_{00}(s) \\ &= \tilde{C}(s)+\tilde{D}(s)\frac{1}{\sqrt{(s+1)(s-1)}}. \end{aligned} \tag{E21}$$

Here

$$\begin{aligned} C(s) &= C_2 s^2 + C_0 \\ &= 2F_2^s s^2+\left(1+\frac{F_1^s}{2}\right)\left[1-\frac{F_2^s}{2}\left(1+2F_0^s\right)\right], \end{aligned} \tag{E22}$$

$$\begin{aligned} D(s) &= D_4 s^4 + D_2 s^2 + D_0 \\ &= 4F_2^s s^4 - 2s^2\left\{F_2^s\left[\left(2+F_0^s\right)\left(1+\frac{F_1^s}{2}\right)-\frac{3}{4}F_1^s\right]-\frac{F_1^s}{2}\right\}+\left[F_2^s\left(1+\frac{3}{2}F_0^s\right)+F_0^s\right]\left(1+\frac{F_1^s}{2}\right), \end{aligned} \tag{E23}$$

and

$$\tilde{C}(s) = C(s)+D(s), \tag{E24}$$

$$\tilde{D}(s) = -sD(s), \tag{E25}$$

where C_0, C_2, D_0, D_2 and D_4 are coefficients that does not depend on s. Thus the secular equation becomes

$$C(s)+D(s)\Pi_{00}(s) = \tilde{C}(s)+\tilde{D}(s)\frac{1}{\sqrt{(s+1)(s-1)}} = 0. \tag{E26}$$

First, we consider the solution $s\to 1+0^+$. When

$$\lim_{s\to 1+0^+}\frac{\tilde{C}(s)}{\tilde{D}(s)} = -1-\frac{F_2^s\left[2-\left(1+\frac{F_1^s}{2}\right)\frac{1}{2}\left(1+2F_0^s\right)\right]+\left(1+\frac{F_1^s}{2}\right)}{F_2^s\left[1-\frac{F_0^s}{2}\left(1+\frac{F_1^s}{2}\right)\right]+F_1^s+F_0^s\left(1+\frac{F_1^s}{2}\right)} \gg 1, \tag{E27}$$

the secular equation has an approximate solution

$$s \simeq 1+\frac{1}{2\left[\frac{\tilde{C}(s=1)}{\tilde{D}(s=1)}\right]^2} = 1+\frac{1}{2\left[1+\frac{F_2^s\left[2-\left(1+\frac{F_1^s}{2}\right)\frac{1}{2}\left(1+2F_0^s\right)\right]+\left(1+\frac{F_1^s}{2}\right)}{F_2^s\left[1-\frac{F_0^s}{2}\left(1+\frac{F_1^s}{2}\right)\right]+F_1^s+F_0^s\left(1+\frac{F_1^s}{2}\right)}\right]^2}. \tag{E28}$$

Second, we look for the solution $s \to +\infty$, which is available when

$$C_2 = \frac{D_4}{2} \tag{E29}$$

$$\frac{\frac{D_0}{2} + \frac{3D_2}{8} + \frac{5D_4}{16}}{C_0 - \frac{D_2}{2} - \frac{3D_4}{8}} = \frac{D_0}{2} + \frac{3D_2}{8} + \frac{5D_4}{16} \gg 1. \tag{E30}$$

In this situation, we have another solution

$$\begin{aligned} s &= \sqrt{\frac{D_0}{2} + \frac{3D_2}{8} + \frac{5D_4}{16}} \\ &= \sqrt{\frac{F_2^s}{8}\left[1 + \left(1 + \frac{F_1^s}{2}\right)\right] + \frac{F_0^s}{2}\left(1 + \frac{F_1^s}{2}\right) + \frac{3F_1^s}{8}}. \end{aligned} \tag{E31}$$

Taking into account of the sufficient condition given in Eq. (E17), we find that the solution will take simpler forms in the two incompressible conditions (i) $1 + \frac{F_1^s}{2} = 0$ and (ii) $F_0^s \to +\infty$:

(i) In the incompressibility condition $1 + \frac{F_1^s}{2} = 0$, we have

$$s = \begin{cases} 1 + \frac{1}{2\left[1 + \frac{2F_2^s}{F_2^s - 2}\right]^2}, & F_2^s > 2, \\ \sqrt{\frac{F_2^s}{8} - \frac{3}{4}}, & F_2^s \to \infty. \end{cases} \tag{E32}$$

When $F_2^s > 2$, there exists at least one weakly damped zero sound mode with the sound speed $c_0 = s v_F = v_F\left(1 + \frac{1}{2\left[1 + \frac{2F_2^s}{F_2^s - 2}\right]^2}\right)$, an additional weakly damped zero sound mode occurs with the sound speed $c_0 = v_F\sqrt{\frac{F_2^s}{8} - \frac{3}{4}}$.

(ii) In the other incompressibility condition $F_0^s \to +\infty$, we have two approximate solutions to the secular equation

$$s = \begin{cases} 1 + \frac{1}{2\left[1 + \frac{2F_2^s}{F_2^s - 2}\right]^2}, & s \to 1 + 0^+, \\ \sqrt{\frac{F_0^s}{2}\left(1 + \frac{F_1^s}{2}\right)}, & s \to \infty. \end{cases} \tag{E33}$$

Here the constraint $F_2^s > 2$ is still imposed by Eq. (E17).

Appendix F: Zero sound: transverse mode in 2D

The algebraic equations type I and II for transverse mode in the collisionless regime in 2D are given by

$$\frac{v_l}{2} + \sum_{l'=0}^{\infty} F_{l'}^s \Xi_{ll'}(s) \frac{v_{l'}}{2} = -\Xi_{l1}(s) U, \tag{F1}$$

and

$$v_l s - \left(1 + \frac{F_{l-1}^s}{2}\right)\frac{v_{l-1}}{2} - \left(1 + \frac{F_{l+1}^s}{2}\right)\frac{v_{l+1}}{2} = \delta_{l2}\frac{U}{2}. \tag{F2}$$

These two algebraic equations are equivalent. To manifest the equivalence, using the following relations

$$\begin{aligned} \Xi_{l2} - 2s\Xi_{l1} =& -\int \frac{d\theta_p}{2\pi} \sin\left(l\theta_p\right) \frac{\cos\theta_p}{s - \cos\theta_p} \sin\left(2\theta_p\right) \\ &+ \int \frac{d\theta_p}{2\pi} \sin\left(l\theta_p\right) \frac{2s\cos\theta_p}{s - \cos\theta_p} \sin\left(\theta_p\right) \\ =& \int \frac{d\theta_p}{2\pi} \sin\left(l\theta_p\right) \sin\left(2\theta_p\right) = \frac{1}{2}\delta_{l2}, \end{aligned}$$

and then multiplying the $l = 1$ and $l = 2$ components of algebraic equation type I by Ξ_{21} and Ξ_{11}, respectively, we can eliminate the U terms and obtain

$$\begin{aligned} 0 =& \left(\frac{v_1}{2} + \sum_{l'=1}^{\infty} F_{l'}^s \Xi_{1l'} \frac{v_{l'}}{2}\right)\Xi_{21} - \left(\frac{v_2}{2} + \sum_{l'=1}^{\infty} F_{l'}^s \Xi_{2l'} \frac{v_{l'}}{2}\right)\Xi_{11} \\ =& \Xi_{21}\frac{v_1}{2} - \Xi_{11}\left(1 + \frac{F_2^s}{2}\right)\frac{v_2}{2} = \Xi_{11}\left[v_1 s - \left(1 + \frac{F_2^s}{2}\right)\frac{v_2}{2}\right], \end{aligned}$$

which is nothing but the $l = 1$ component of algebraic equation of type II. Note here we have used that $\Xi_{l0}(s) = 0$.

1. Two channel model

Now we consider a two channel model by keeping only F_1^s and F_2^s and setting $F_l^s = 0$ for $l \geq 3$. The components of algebraic equation I can be classified into two categories: (i) $l \geq 3$ and (ii) $l = 1$ and $l = 2$. The components in the second category form a close set that is composed of v_1 and v_2:

$$\frac{v_1}{2} + F_1^s \Xi_{11} \frac{v_1}{2} + F_2^s \Xi_{12} \frac{v_2}{2} = -\Xi_{11} U, \tag{F3}$$

$$\frac{v_2}{2} + F_1^s \Xi_{21} \frac{v_1}{2} + F_2^s \Xi_{22} \frac{v_2}{2} = -\Xi_{21} U. \tag{F4}$$

The solution from algebraic equation I leads to the response functions as follows,

$$\frac{v_1}{2U} = -\frac{\left(1 + \frac{F_2^s}{2}\right)\Xi_{11}}{\left(1 + F_1^s \Xi_{11}\right)\left(1 + \frac{F_2^s}{2}\right) + F_2^s 4s^2 \Xi_{11}}, \tag{F5}$$

$$\frac{v_2}{2U} = -\frac{2s\Xi_{11}}{\left(1 + F_1^s \Xi_{11}\right)\left(1 + \frac{F_2^s}{2}\right) + F_2^s 4s^2 \Xi_{11}}. \tag{F6}$$

The zero sound mode frequency can be determined via the pole of the response functions, i.e.,

$$\left(1 + F_1^s \Xi_{11}\right)\left(1 + \frac{F_2^s}{2}\right) + F_2^s 4s^2 \Xi_{11} = 0. \tag{F7}$$

To find a real solution s to the above equation, we define a function

$$\begin{aligned} \bar{h}_2(s) =& \left(1 + \frac{F_2^s}{2}\right) + \Xi_{11}\left[F_1^s\left(1 + \frac{F_2^s}{2}\right) + F_2^s 4s^2\right] \\ =& \left(1 + \frac{F_2^s}{2}\right) + \frac{1 - 2s^2}{2}\Pi_{00}\left[F_1^s\left(1 + \frac{F_2^s}{2}\right) + F_2^s 4s^2\right], \end{aligned} \tag{F8}$$

we have $\bar{h}_2(s\to\infty) = 2F_2^s s^2$ and meanwhile

$$\bar{h}_2(s\to 1) = \frac{1}{2}\frac{F_1^s\left(1+\frac{F_2^s}{2}\right)+4F_2^s}{\sqrt{2(s-1)}} + O(1). \quad \text{(F9)}$$

To have a real solution for $\bar{h}_2(s) = 0$, one requires

$$F_2^s\left[F_1^s\left(1+\frac{F_2^s}{2}\right)+4F_2^s\right] < 0. \quad \text{(F10)}$$

Under the condition of QSL in 2D $1+\frac{F_1^s}{2} = 0$, it reduces to

$$F_2^s\left(3F_2^s - 2\right) < 0 \quad \text{(F11)}$$

Thus we have transverse mode for $0 < F_2^s < 2/3$ under the condition of QSL in 2D.

Eq. (**??**) must generally be found numerically. In the limit $0 < s-1 \ll 1$, we can obtain the approximately analytical solution

$$s = 1 + \frac{1}{2\left[1 - \frac{2\left(1+\frac{F_2^s}{2}\right)}{F_1^s\left(1+\frac{F_2^s}{2}\right)+4F_2^s}\right]^2}. \quad \text{(F12)}$$

Under the condition of QSL in 2D $1+\frac{F_1^s}{2} = 0$, it simplifies to

$$s = 1 + \frac{1}{8}\left(\frac{3F_2^s - 2}{F_2^s - 2}\right)^2. \quad \text{(F13)}$$

Appendix G: First sound: longitudinal mode in 2D

In 2D, we assume that the collision integral takes the form of

$$I\left[n_\mathrm{p}\right] = -\frac{1}{\tau}\left[\delta n_\mathrm{p} - \left\langle\delta n_\mathrm{p}\right\rangle - 2\left\langle\delta n_\mathrm{p}\cos\theta_\mathrm{p}\right\rangle\cos\theta_\mathrm{p}\right]. \quad \text{(G1)}$$

The density fluctuation is given by

$$\delta n_\mathrm{p} = -\frac{\partial n_\mathrm{p}^0}{\partial\varepsilon_\mathrm{p}}\nu_\mathrm{p}, \quad \text{(G2)}$$

where ν_p can be expanded in 2D as follows

$$\nu_\mathrm{p} = \sum_{l=0}^{\infty}\cos\left(l\theta_\mathrm{p}\right)\nu_l. \quad \text{(G3)}$$

The expectation values in the collision integral $I[n_\mathrm{p}]$ can be computed as

$$\left\langle\delta n_\mathrm{p}\right\rangle = -\frac{\partial n_\mathrm{p}^0}{\partial\varepsilon_\mathrm{p}}\nu_0, \quad \text{(G4)}$$

$$2\left\langle\delta n_\mathrm{p}\cos\theta_\mathrm{p}\right\rangle = -\frac{\partial n_\mathrm{p}^0}{\partial\varepsilon_\mathrm{p}}\nu_1. \quad \text{(G5)}$$

The Landau kinetic equation [Eq. (**??**)] in the collision regime reads

$$\frac{\partial\delta n_\mathrm{p}}{\partial t} + \vec{v}_\mathrm{p}\cdot\nabla_\mathrm{r}\left(\delta n_\mathrm{p} - \frac{\partial n_\mathrm{p}^0}{\partial\varepsilon_\mathrm{p}}\delta\varepsilon_\mathrm{p}\right) = I[n_\mathrm{p}].$$

Taking into account of a scalar external field $U(q,\omega) = Ue^{i(q\cdot r-\omega t)}$, in the collisionless regime, the quasiparticle excitation energy reads

$$\delta\varepsilon_\mathrm{p} = U + \sum_{\mathrm{p}'} f_{\mathrm{pp}'}^s\delta n_{\mathrm{p}'}, \quad \text{(G6)}$$

and the Landau kinetic equation can be rewritten in frequency-momentum space as

$$\begin{aligned}&\left(\omega + \frac{i}{\tau} - q\cdot\vec{v}_\mathrm{p}\right)\nu_\mathrm{p} + \left(q\cdot\vec{v}_\mathrm{p}\right)\left(-U + \sum_{\mathrm{p}'} f_{\mathrm{pp}'}^s\frac{\partial n_{\mathrm{p}'}^0}{\partial\varepsilon_{\mathrm{p}'}}\nu_{\mathrm{p}'}\right)\\ &= \frac{i}{\tau}\left(\nu_0 + \nu_1\cos\theta_\mathrm{p}\right).\end{aligned} \quad \text{(G7)}$$

Putting the expansion of ν_p into the above equation, we obtain reduced Landau kinetic equation in the $U = 0$ limit:

$$\begin{aligned}&\sum_{l=0}^{\infty}\cos\left(l\theta_\mathrm{p}\right)\nu_l - \frac{q\cdot\vec{v}_\mathrm{p}}{\omega + \frac{i}{\tau} - q\cdot\vec{v}_\mathrm{p}}\sum_{l=0}^{\infty}F_l^s\cos\left(l\theta_\mathrm{p}\right)\frac{\nu_l + \delta_{l0}\nu_0}{2}\\ &= \frac{1}{\omega + \frac{i}{\tau} - q\cdot\vec{v}_\mathrm{p}}\frac{i}{\tau}\left(\nu_0 + \nu_1\cos\theta_\mathrm{p}\right).\end{aligned}$$

Using the orthogonal relations for cosine functions,

$$\int\frac{d\theta_\mathrm{p}}{2\pi}\cos\left(l\theta_\mathrm{p}\right)\cos\left(l'\theta_\mathrm{p}\right) = \frac{1}{2}\left(\delta_{ll'} + \delta_{l0}\delta_{l'0}\right),$$

we obtain the algebraic equation I in the collision regime as

$$\begin{aligned}&\frac{\nu_l + \delta_{l0}\nu_0}{2} + \sum_{l'=0}^{\infty}F_{l'}^s\Pi_{ll'}(\tilde{s})\frac{\nu_{l'} + \delta_{l'0}\nu_0}{2}\\ &= -i\kappa\left[\Pi_{l0}(\tilde{s})\nu_1 + \Pi_l(\tilde{s})\nu_0\right],\end{aligned} \quad \text{(G8)}$$

where

$$s = \frac{\omega}{qv_F},\ \kappa = \frac{1}{\tau qv_F} = \frac{s}{\omega\tau},\ \tilde{s} = \frac{\omega}{qv_F} + \frac{i}{\tau qv_F} = s + i\kappa,$$

and

$$\begin{aligned}\Pi_{ll'}(\tilde{s}) &= -\int\frac{d\theta_\mathrm{p}}{2\pi}\cos\left(l\theta_\mathrm{p}\right)\frac{\cos\theta_\mathrm{p}}{\tilde{s} - \cos\theta_\mathrm{p}}\cos\left(l'\theta_\mathrm{p}\right),\\ \Pi_l(\tilde{s}) &= \frac{1}{\tilde{s}}\left(\Pi_{l0}(\tilde{s}) - \delta_{l0}\right).\end{aligned}$$

Indeed, the algebraic equation type I now contains κ-terms which are induced by collisions.

1. Two channel model

To study first sound mode, we consider a two channel model by keeping F_0^s and F_1^s and setting $F_l^s = 0$ for $l \geq 2$. Then Landau kinetic equation becomes

$$\begin{pmatrix} 1 + F_0^s\Pi_{00} + i\kappa\Pi_0 & \frac{1}{2}F_1^s\Pi_{01} + i\kappa\Pi_{00} \\ F_0^s\Pi_{10} + i\kappa\Pi_1 & \frac{1}{2}\left(1 + F_1^s\Pi_{11}\right) + i\kappa\Pi_{10} \end{pmatrix}\begin{pmatrix} \nu_0 \\ \nu_1 \end{pmatrix} = 0. \tag{G9}$$

The condition for a nontrivial solution allows us to determine the frequency and leads to

$$\begin{aligned} &\det\begin{pmatrix} 1 + F_0^s\Pi_{00} + i\kappa\Pi_0 & \frac{1}{2}F_1^s\Pi_{01} + i\kappa\Pi_{00} \\ F_0^s\Pi_{10} + i\kappa\Pi_1 & \frac{1}{2}\left(1 + F_1^s\Pi_{11}\right) + i\kappa\Pi_{10} \end{pmatrix} \\ &= \frac{1}{2}\Pi_{00}\left[\left(F_0^s + \frac{i\kappa}{\tilde{s}}\right)\left(1 + \frac{F_1^s}{2}\right) + 2\tilde{s}^2\left(1 - \frac{i\kappa}{\tilde{s}}\right)\left(\frac{F_1^s}{2} + \frac{i\kappa}{\tilde{s}}\right)\right] \\ &\quad + \frac{1}{2}\left(1 - \frac{i\kappa}{\tilde{s}}\right)\left(1 + \frac{F_1^s}{2}\right) = 0, \end{aligned}$$

where we have used $\Pi_{10}(\tilde{s}) = \tilde{s}\Pi_{00}(\tilde{s})$, $\Pi_{11}(\tilde{s}) = \tilde{s}^2\Pi_{00}(\tilde{s}) + \frac{1}{2}$, $\Pi_0(\tilde{s}) = \frac{1}{\tilde{s}}(\Pi_{00}(\tilde{s}) - 1)$, and $\Pi_1(\tilde{s}) = \frac{1}{\tilde{s}}\Pi_{10}(\tilde{s}) = \Pi_{00}(\tilde{s})$. In the hydrodynamic limit of $\omega\tau \ll 1$, we have $\tilde{s} \to i\infty$ and

$$\Pi_{00}(\tilde{s}) = 1 - \frac{\tilde{s}}{\sqrt{\tilde{s}^2 - 1}} \approx -\frac{1}{2\tilde{s}^2} - \frac{3}{8\tilde{s}^4}. \tag{G10}$$

To the first order of $i\omega\tau$, we have

$$1 - \frac{i\kappa}{\tilde{s}} = \frac{s}{\tilde{s}}$$

and

$$\frac{i\kappa}{\tilde{s}} = \frac{1}{1 - i\omega\tau} \approx 1 + i\omega\tau.$$

Thus in the hydrodynamic limit, we obtain

$$\begin{aligned} &\Pi_{00}\left[\left(F_0^s + \frac{i\kappa}{\tilde{s}}\right)\left(1 + \frac{F_1^s}{2}\right) + 2\tilde{s}^2\left(1 - \frac{i\kappa}{\tilde{s}}\right)\left(\frac{F_1^s}{2} + \frac{i\kappa}{\tilde{s}}\right)\right] \\ &+ \left(1 - \frac{i\kappa}{\tilde{s}}\right)\left(1 + \frac{F_1^s}{2}\right) \\ &\approx \frac{1}{\tilde{s}^2}\left[s^2 - \frac{1}{2}\left(1 + F_0^s\right)\left(1 + \frac{F_1^s}{2}\right) + \frac{1}{4}i\omega\tau\left(1 + \frac{F_1^s}{2}\right)\right] = 0. \end{aligned}$$

The dispersion relation between ω and q for first sound in 2D now reads

$$\left(\frac{\omega}{qv_F}\right)^2 = \frac{1}{2}\left(1 + F_0^s\right)\left(1 + \frac{F_1^s}{2}\right) - \frac{1}{4}i\omega\tau\left(1 + \frac{F_1^s}{2}\right). \tag{G11}$$
